# Impaired liver-muscle lactate metabolism causes sarcopenia via lactic acidosis in skeletal muscle in mice

**DOI:** 10.1126/sciadv.aeb4011

**Published:** 2026-09-04

**Authors:** Takahiro Eguchi, Nozomi Hayashiji, Sho Tabata, Keiko Kabetani, Yasunori Shintani, Hideki Makinoshima, Naoki Ito

**Affiliations:** ^1^ Brain-Skeletal Muscle Connection in Aging Project Team, Geroscience Research Center, National Center for Geriatrics and Gerontology, Obu, Japan.; ^2^ Juntendo Advanced Research Institute for Health Science, Juntendo University Graduate School of Medicine, Tokyo, Japan.; ^3^ Tsuruoka Metabolomics Laboratory, National Cancer Center, Tsuruoka, Japan.; ^4^ Shonai Regional Industry Promotion Center, Tsuruoka, Japan.; ^5^ Division of Translational Informatics, Exploratory Oncology Research and Clinical Trial Center, National Cancer Center, Kashiwa, Japan.; ^6^ Department of Molecular Pharmacology, National Cerebral and Cardiovascular Center, Suita, Osaka, Japan.

## Abstract

Sarcopenia is a progressive disease characterized by age-related decline in skeletal muscle force and mass. The fundamental molecular pathogenesis of sarcopenia has not yet been elucidated. Here, we show that the accumulation of lactate and intracellular acidification, lactic acidosis, in skeletal muscle owing to impaired liver-skeletal muscle lactate metabolism is the fundamental cause of sarcopenia. Systemic lactate tolerance decreased in aged mice owing to the impaired lactate processing capacity in the liver, which caused lactic acidosis in skeletal muscle. Furthermore, pharmacological activation of hypoxia-inducible factor (HIF) or liver-specific activation of HIF1α improved age-associated impairment in lactate tolerance, lactic acidosis in skeletal muscle, and sarcopenia. Mechanistically, the decreased nicotinamide adenine dinucleotide level was the cause of dysregulated skeletal muscle functions due to lactic acidosis. Using mouse models, our results show lactic acidosis in skeletal muscle as a key molecular pathogenesis of sarcopenia and highlight HIF1α in the liver as a pharmacological target for sarcopenia.

## INTRODUCTION

Skeletal muscle, the largest organ, constituting approximately 40 percent of our body, is essential for physical functions (*1*). The age-related loss of muscle force and mass, sarcopenia, is a geriatric syndrome associated with adverse health outcomes in older adults, leading to decreased quality of life and increased healthcare costs and mortality (*2*). In addition to physical functions, skeletal muscle has a key role in metabolic homeostasis via its endocrine cross-talk with other organs (*3*). Recent studies have suggested that dysfunction in other tissues, such as the liver, is one of the causes of age-related loss of muscle force and mass (*4*, *5*). Indeed, patients with hepatic insufficiency have a high prevalence of sarcopenia (*4*, *5*). Age-related dysfunction in inter-organ communication is involved in the pathogenesis of sarcopenia, although its underlying mechanisms, responsible metabolic pathway, and metabolites remain largely unknown.

Importantly, loss of muscle force precedes loss of muscle mass, and a reduction in muscle force is associated with mortality in older adults (*6*). Dysfunction of mitochondria is one of the causes of decreased muscle force in sarcopenia (*7*, *8*). Mitochondrial homeostasis depends on nicotinamide adenine dinucleotide (NAD^+^). NAD^+^ is a cofactor for multiple metabolic pathways, and is required for NAD^+^-consuming enzymes such as sirtuins, mammalian NAD^+^-dependent protein deacetylases (*9*–*12*). The progressive and gradual decreases in NAD^+^ levels and the resultant dysfunctions of NAD^+^-consuming enzymes are a driving force of age-associated pathophysiologies (*9*–*11*). In older adults with sarcopenia, both mitochondrial function and NAD^+^ levels are reduced in skeletal muscle, suggesting that dysregulated NAD^+^ metabolism is involved in mitochondrial dysfunction in sarcopenia (*7*). However, the upstream causes of the reduction in skeletal muscle NAD^+^ levels during aging remain unknown. A recent report has suggested that a decline in skeletal muscle NAD^+^ is not involved in skeletal muscle dysfunction (*13*). The role of skeletal muscle NAD^+^ in the pathogenesis of sarcopenia remains controversial.

Several factors, including impaired inter-organ communication, mitochondrial dysfunction, and metabolic changes during aging, are thought to contribute to the pathogenesis of sarcopenia. However, it is unclear how these dysfunctions are integrated to lead to sarcopenia. The underlying molecular mechanisms and metabolic pathways directly involved in the pathogenesis of sarcopenia have not yet been identified, which impedes the development of therapeutic strategies for sarcopenia. To elucidate the underlying pathogenesis of sarcopenia, we sought to characterize age-related muscle phenotypes and isolate the key molecular drivers of these alterations.

## RESULTS

### Identification and characterization of the Fluo-4 abnormal muscle fibers in aged mice

**Fig. 1.**
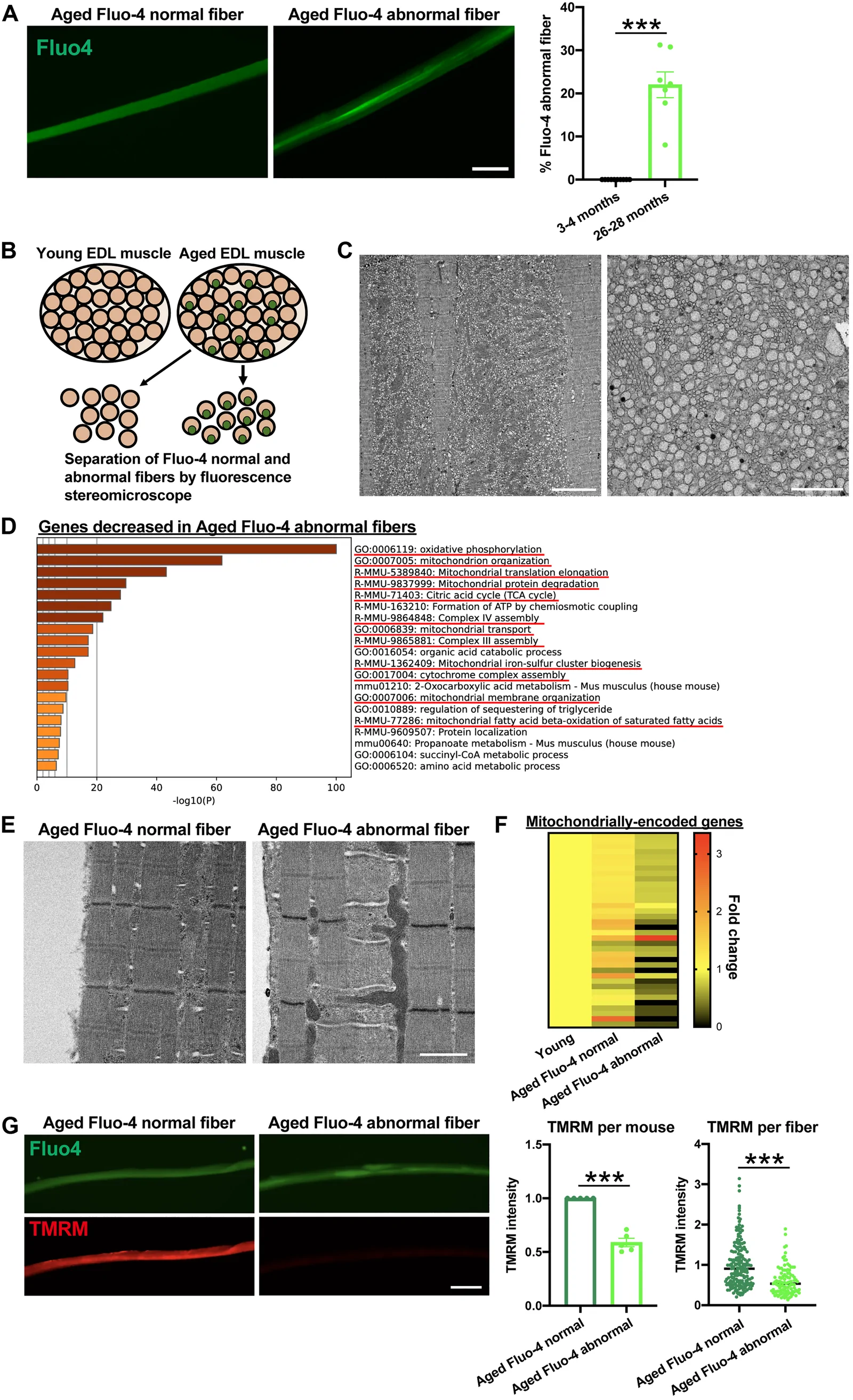
Identification and characterization of the Fluo-4 abnormal muscle fibers in aged mice. (**A**) Left: representative fluorescence image of the Fluo-4 normal and Fluo-4 abnormal muscle fibers in 26–28-month-old aged mice. Bar: 100 μm. Right: quantitative analysis of the Fluo-4 abnormal fibers in 3–4-month-old young mice and 26–28-month-old mice. *n* = 7–10. (**B**) Schematic images for the separation of the Fluo-4 normal and abnormal fibers from 26–28-month-old mice. (**C**) Representative ultrastructure of tubular aggregate-like structures in the Fluo-4 abnormal fibers. Bar: 5 μm (left) and 1 μm (right). *n* = 4. (**D**) Gene ontology analysis of genes decreased in the Fluo-4 abnormal fibers compared with the Fluo-4 normal fibers. (**E**) Representative ultrastructure of morphologically abnormal mitochondria in the Fluo-4 abnormal fibers. Bar: 1 μm. *n* = 4. (**F**) Heat map analysis of the expression of mitochondrially-encoded genes. (**G**) Left: representative fluorescence image of TMRM in the Fluo-4 normal and abnormal fibers. Middle and right: quantitative analysis of TMRM intensity in the Fluo-4 normal and abnormal fibers per mouse (middle) and per fiber (right). Bar: 100 μm. *n* = 5. ***P < 0.001 by Student’s t-test. Error bars indicate s.e.m.

In previous studies, we analyzed intracellular Ca^2+^ levels in single fibers from the extensor digitorum longus (EDL) muscles using the fluorescent Ca^2+^ indicator Fluo-4 (*14*–*16*). Interestingly, we discovered the presence of aberrant Fluo-4 staining in some single muscle fibers from 26–28-month-old aged male mice (Fig. 1A). Approximately 20% of single muscle fibers showed abnormal, apparently higher Fluo-4 fluorescence (Fig. 1A). This abnormal Fluo-4 staining was not observed in single muscle fibers from 3–4-month-old young mice (Fig. 1A). To characterize the Fluo-4 abnormal fibers in 26–28-month-old mice, we co-stained these abnormal fibers with the ER-tracker, a marker of sarcoplasmic reticulum (SR) in skeletal muscle, and Mitotracker, a marker of mitochondria. The intense Fluo-4 fluorescence was co-stained with the ER-tracker but not with Mitotracker, suggesting the association of abnormal Fluo-4 staining with SR (fig. S1, A and B). In addition, Calcein-AM was distributed uniformly throughout the single muscle fiber and did not accumulate in any particular regions, suggesting that the high fluorescence intensity of Fluo-4 or ER-tracker was not due to local dye accumulation (fig. S1C).

To further characterize the Fluo-4 abnormal fibers observed during aging, we separated the Fluo-4 normal and Fluo-4 abnormal single muscle fibers from 26–28-month-old mice using a fluorescence stereomicroscope (Fig. 1B) and analyzed their ultrastructure by electron microscopy. The Fluo-4 abnormal fibers had an ultrastructure similar to that of a tubular aggregate, with regular arrays of tubules derived from SR (Fig. 1C) (*17*). The number of tubular aggregate-positive fibers increases during aging only in male mice (*18*). Consistently, the Fluo-4 abnormal fibers were hardly detected in 26–28-month-old female mice (only one abnormal fiber was observed in 373 fibers). Furthermore, the Fluo-4 abnormal fibers showed abnormal localization of the ryanodine receptor (RyR), dihydropyridine receptor (DHPR), and sarcoplasmic/endoplasmic reticulum Ca^2+^-ATPase (SERCA), markers of SR (fig. S1, D to F). This abnormal localization of RyR, DHPR, and SERCA was not observed in the Fluo-4 normal fibers (fig. S1, D to F). Abnormal localization of RyR, DHPR, or SERCA was also detected in the transverse muscle sections from 18–20-month-old middle-aged mice (fig. S1G). Furthermore, caffeine-induced increases in intracellular Ca^2+^ levels were attenuated in the Fluo-4 abnormal fibers, suggesting impaired Ca^2+^ handling (fig. S1H). These results clearly revealed the presence of the Fluo-4 abnormal, tubular aggregate-like structure-positive muscle fibers in aged mice. Because the number of these abnormal skeletal muscle fibers significantly increased during aging, they provided an opportunity to identify the molecular changes potentially associated with sarcopenia.

### Mitochondrial abnormalities in the Fluo-4 abnormal muscle fibers in aged mice

To characterize the molecular signature of these Fluo-4 abnormal fibers in 26–28-month-old mice, we separated the Fluo-4 normal and abnormal fibers and performed RNA-seq analysis. Gene ontology analysis revealed that SR-related ontology, such as protein processing in endoplasmic reticulum or protein localization to endoplasmic reticulum, were upregulated in the Fluo-4 abnormal fibers compared with the Fluo-4 normal fibers (fig. S1I), consistent with the SR-enriched tubular aggregate-like structures in the Fluo-4 abnormal fibers.

We also found that genes related to oxidative phosphorylation, tricarboxylic acid (TCA) cycle, and mitochondria were downregulated in the Fluo-4 abnormal fibers compared with the Fluo-4 normal fibers (Fig. 1D). Consistently, we observed abnormally swollen mitochondria in the Fluo-4 abnormal fibers (Fig. 1E). In addition, the Fluo-4 abnormal fibers showed decreased expression of mitochondrially-encoded genes, the genes encoding the proteins and RNA crucial for mitochondrial functions, which are encoded by the mitochondrial genome (Fig. 1F and fig. S1J) (*19*). To analyze mitochondrial activity in the Fluo-4 abnormal fibers, we stained them with tetramethylrhodamine methylester (TMRM), a fluorescent probe for mitochondrial membrane potential. We found weaker TMRM signals in the Fluo-4 abnormal fibers than those in the Fluo-4 normal fibers (Fig. 1G), suggesting decreased mitochondrial activity in the Fluo-4 abnormal fibers. Mitochondrial abnormalities in skeletal muscle during aging are well-known (*7*). Nonetheless, at the single muscle fiber level, age-dependent mitochondrial abnormalities are more prominent in the Fluo-4 abnormal fibers than in the Fluo-4 normal fibers, suggesting that mitochondrial dysfunction does not occur uniformly throughout skeletal muscle during aging and that specific molecular abnormalities occur in the Fluo-4 abnormal fibers.

### *Ckmt2* and *Perm1* as the genes responsible for decreased mitochondrially-encoded genes in the Fluo-4 abnormal fibers in aged mice and age-associated decline in muscle force

**Fig. 2.**
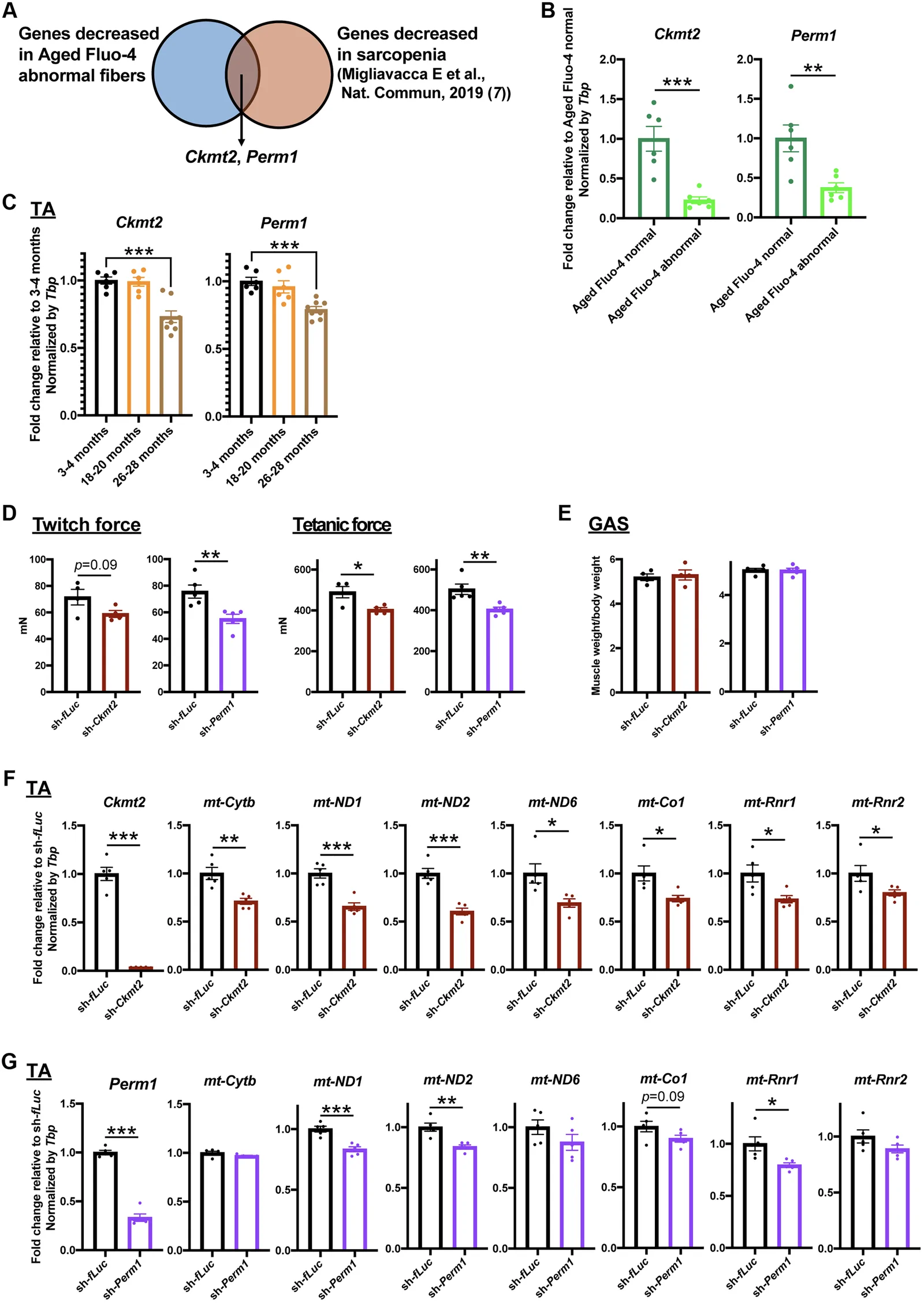
*Ckmt2* and *Perm1* as the responsible genes for mitochondrially-encoded genes and age-associated decline in muscle force. (**A**) Schematic illustration of common genes between those downregulated in the Fluo-4 abnormal fibers and those downregulated in older adults with sarcopenia. (**B**) mRNA expression levels of *Ckmt2* and *Perm1* in the Fluo-4 normal and abnormal fibers. *n* = 6. (**C**) mRNA expression levels of *Ckmt2* and *Perm1* in the TA muscle of 3–4-month-old young, 18–20-month-old middle-aged and 26–28-month-old aged mice. *n* = 6–8. (**D**) Twitch and tetanic force measurements of *Ckmt2*- or *Perm1*-knockdown mice. *n* = 4–5. (**E**) The GAS muscle weights of *Ckmt2*- or *Perm1*-knockdown mice. *n* = 4–5. (**F** and **G**) mRNA expression levels of *Ckmt2*, *Perm1* and mitochondrially-encoded genes in *Ckmt2*-knockdown mice (F) and *Perm1*-knockdown mice (G). *n* = 5. *P < 0.05, **P < 0.01, and ***P < 0.001 by Student’s t-test for (B), (D), (F), and (G), or by one-way ANOVA with Dunnett’s test for (C). Error bars indicate s.e.m.

To identify the specific molecular changes responsible for mitochondrial abnormalities, including the downregulation of mitochondrially-encoded genes, in the Fluo-4 abnormal fibers, we particularly focused on genes downregulated in the Fluo-4 abnormal fibers compared with those in the Fluo-4 normal fibers (Fig. 2A). In addition, we analyzed the human RNA-seq data reported by Migliavacca *et al.* (*7*)., which compared healthy older adults and older adults with sarcopenia. Focusing on the common genes between these two data sets, we obtained two candidates, *creatine kinase, mitochondrial 2* (*Ckmt2*), and *PPARGC1 and ESRR induced regulator, muscle 1* (*Perm1*). Ckmt2 is localized at the intermembrane space of mitochondria, and is a key regulator of oxidative phosphorylation and mitochondrial respiration in skeletal muscle (*20*, *21*). *Ckmt2* was listed as the 78th gene from the top, with a high negative coefficient of association with sarcopenia (*7*). Perm1 is an upstream regulator of peroxisome proliferator-activated receptor γ coactivator 1α (PGC-1α), and enhances mitochondrial biogenesis and oxidative metabolism in skeletal muscle (*22*, *23*). *Perm1* was listed as the 514th from the top, with a negative coefficient of association with sarcopenia (*7*). *Ckmt2* and *Perm1* were highly expressed in skeletal and heart muscle compared with other tissues (fig. S2, A and B). These genes were significantly downregulated in the Fluo-4 abnormal fibers compared with the Fluo-4 normal fibers (Fig. 2B). Furthermore, the expression of *Ckmt2* and *Perm1* was decreased in the skeletal muscle of 26–28-month-old mice compared with that of young mice (Fig. 2C).

Remarkably, knockdown of *Ckmt2* or *Perm1* in the gastrocnemius (GAS) muscle of young mice led to reduced tetanic force, compared to control (Fig. 2D). In addition to tetanic force, knockdown of *Perm1* resulted in reduced twitch force (Fig. 2D). We also observed reduced specific force following knockdown of *Ckmt2* or *Perm1* (fig. S2C). There were no differences in 100% contraction time or 50% relaxation time (fig. S2, D and E). In contrast, knockdown of *Cktm2* or *Perm1* did not affect muscle weight (Fig. 2E), indicating that *Ckmt2* and *Perm1* are required for the maintenance of muscle force but not muscle mass. To investigate the involvement of *Ckmt2* and *Perm1* in the decreased expression of mitochondrially-encoded genes in the Fluo-4 abnormal fibers, we analyzed the expression of mitochondrially-encoded genes. We used the tibialis anterior (TA) muscle, and the knockdown efficiencies of sh-*Ckmt2* and sh-*Perm1* were 97% and 67%, respectively. Intriguingly, knockdown of *Ckmt2* in the TA muscle led to downregulation of mitochondrially-encoded genes (Fig. 2F). Although less effective than *Ckmt2*, knockdown of *Perm1* also led to downregulation of *mt-ND1*, *mt-ND2*, and *mt-Rnr1* (Fig. 2G). In addition to in vivo knockdown analyses, knockdown of *Ckmt2* or *Perm1* in cultured primary myotubes also showed downregulation of *mt-Cytb*, *mt-ND1*, and *mt-ND2* (fig. S2F), indicating the reduced expression of *Ckmt2* and *Perm1* as a cause of downregulation of mitochondrially-encoded genes in the Fluo-4 abnormal fibers. These results demonstrate that *Ckmt2* and *Perm1* are key regulators of muscle force, but not muscle mass, indicating that reduced muscle force and mitochondrial dysfunction in sarcopenia are likely induced by the downregulation of *Ckmt2* and *Perm1*.

### Lactic acidosis in skeletal muscle as a cause of age-related decline in muscle force

**Fig. 3.**
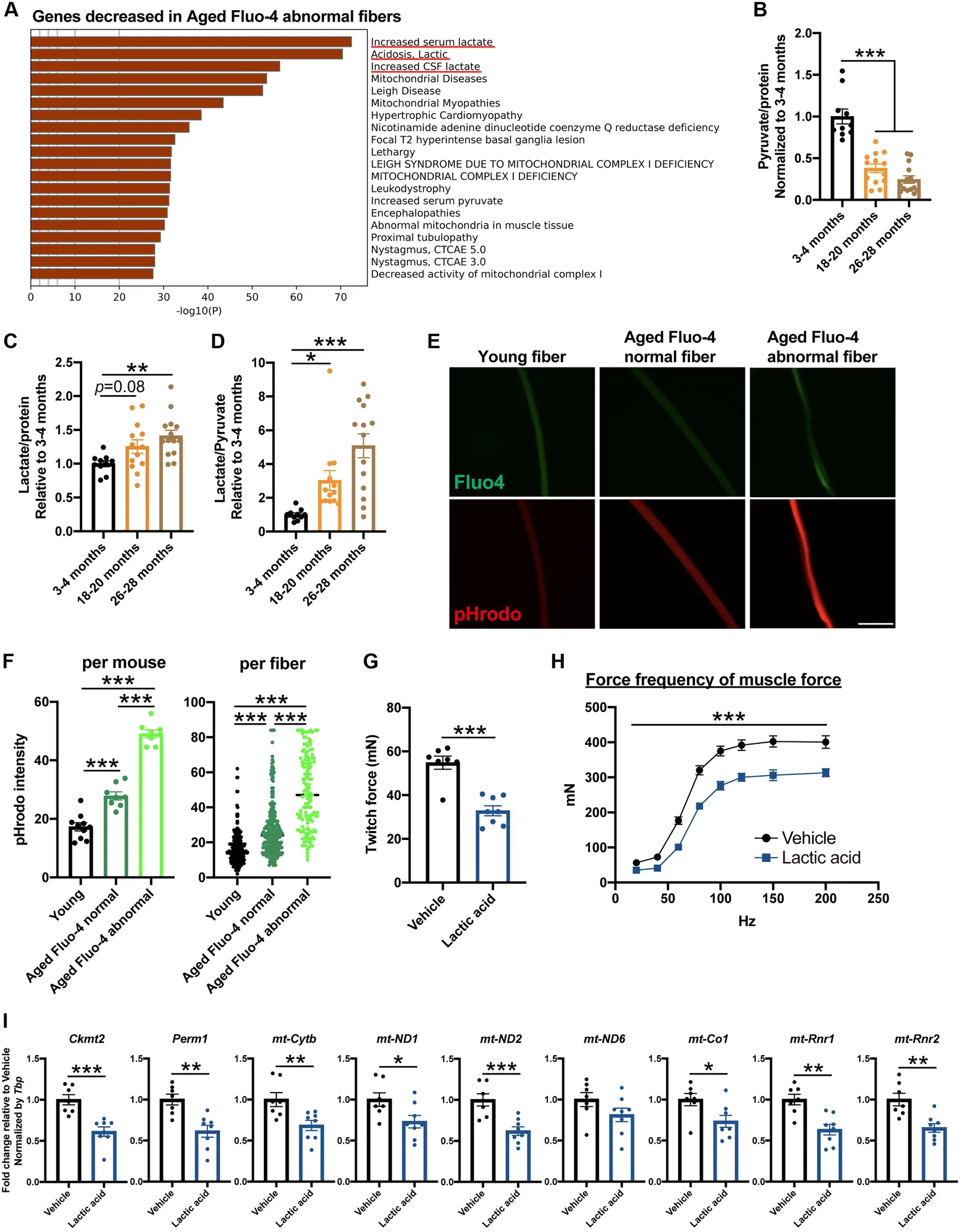
Lactic acidosis in skeletal muscle as a cause of age-related decline in muscle force. (**A**) Gene-disease network analysis showed that the gene set downregulated in the aged Fluo-4 abnormal fibers is significantly associated with those altered during lactic acidosis. (**B** to **C**) Amounts of pyruvate (B), lactate (C), or lactate/pyruvate ratio (**D**) in the TA muscle of 3–4-month-old young, 18–20-month-old middle-aged, and 26–28-month-old aged mice. *n* = 10–14. (**E**) Representative fluorescence image of pHrodo in single muscle fibers from young mice, Fluo-4 normal fibers, or Fluo-4 abnormal fibers from 26–28-month-old mice. Bar: 100 μm. (**F**) Quantitative analysis of pHrodo intensity in single muscle fibers from young mice, Fluo-4 normal fibers or Fluo-4 abnormal fibers from 26–28-month-old mice per mouse (left) and per fiber (right). *n* = 8–10. (**G**) Twitch force measurement after administration of lactic acid. *n* = 7–8. (**H**) In vivo muscle force-frequency curve of lactic acid-administered mice. *n* = 7–8. (**I**) mRNA expression levels of *Ckmt2*, *Perm1,* and mitochondrially-encoded genes in lactic acid-administered mice. *n* = 7–8. *P < 0.05, **P < 0.01 and ***P < 0.001 by Student’s t-test for (G) and (I), one-way ANOVA with Dunnett’s test for (B), (C) and (D), by one-way ANOVA with Tukey’s test for (F), or by two-way repeated-measures ANOVA for (H). Error bars indicate s.e.m.

We then attempted to identify the upstream causes of abnormalities that occurred in the Fluo-4 abnormal fibers in aged mice. We performed a gene-disease network analysis using Metascape (*24*) and DisGeNET (*25*) to explore the diseases that showed gene expression changes similar to those occurring in the Fluo-4 abnormal fibers. Because of the mitochondrial abnormalities in the Fluo-4 abnormal fibers, we suspected that there were high similarities between the gene expression profiles of the Fluo-4 abnormal fibers and those of mitochondrial disease or mitochondrial myopathies. Unexpectedly, the highest similarity was observed with lactic acidosis, such as “Increased serum lactate,” “Acidosis, Lactic,” and “Increased CSF lactate” (Fig. 3A). In addition to the Fluo-4 abnormal fibers, we performed the same analysis using the genes with a negative coefficient of association with sarcopenia reported by Migliavacca *et al.* (*7*). Similar to the Fluo-4 abnormal fibers, the genes with a negative coefficient of association with sarcopenia showed a high similarity with lactic acidosis (fig. S3A), suggesting the involvement of lactic acidosis in sarcopenia both in mice and humans.

Lactate is one of the classical byproducts of glycolysis (*26*). Lactate is produced from pyruvate, an end product of glycolysis, by lactate dehydrogenase A (LDHA) (*27*). Lactic acidosis is a metabolic acidosis resulting from increased levels of lactic acid and intracellular acidification (*28*). From our gene-disease network analysis, we hypothesized that lactic acidosis is involved in the pathogenesis of sarcopenia. To explore this possibility, we analyzed the changes in lactate levels in skeletal muscle during aging. In our previous study, we demonstrated a gradual decrease in pyruvate levels in skeletal muscle during aging, suggesting decreased glycolysis in skeletal muscle during aging (*29*). Similar to our previous observation, pyruvate levels were decreased in the TA muscle of aged mice (Fig. 3B). On the other hand, lactate levels were increased in the TA muscle of aged mice (Fig. 3C). Because of decreased pyruvate and increased lactate levels, the lactate/pyruvate ratio was apparently higher in 18–20-month-old and 26–28-month-old mice than in young mice (Fig. 3D). To confirm acidosis in aged skeletal muscle, we stained single muscle fibers with pHrodo, a pH indicator. A decrease in pH, an intracellular acidification, results in increased fluorescence of pHrodo. We found that the Fluo-4 normal fibers from 26–28-month-old mice showed much higher pHrodo intensity than those from young mice (Fig. 3, E and F). Furthermore, the Fluo-4 abnormal fibers showed much greater fluorescence compared with the Fluo-4 normal fibers (Fig. 3, E and F). Although there was a significant difference in pHrodo fluorescence, no such difference was observed in Fluo-4 intensity within the normal regions between the aged Fluo-4 normal fibers and abnormal fibers (fig. S3B). Importantly, these alterations in pHrodo fluorescence fell within the physiological range of pH variations (fig. S3C). These results clearly indicate the occurrence of lactic acidosis in aged skeletal muscle, and intracellular acidification is more prominent in the Fluo-4 abnormal fibers. Although 26–28-month-old female mice did not have the Fluo-4 abnormal fibers, pHrodo intensity was much higher in single muscle fibers from 26–28-month-old female mice compared with those from young female mice (fig. S3D), suggesting intracellular acidification as a sex-independent phenomenon occurring during aging.

To examine whether lactic acidosis indeed affects muscle force and the *Ckmt2*/*Perm1*-mitochondrially-encoded genes axis, we performed an intramuscular injection of lactic acid in young mice. We observed a decline in twitch and tetanic forces, but not in the GAS muscle weight, after the administration of lactic acid (Fig. 3, G and H and fig. S3E). We also observed a decline in specific force, but no changes in 100% contraction time or 50% relaxation time (fig. S3, F to H). Furthermore, the expression of *Ckmt2*, *Perm1*, and mitochondrially-encoded genes also decreased after the administration of lactic acid (Fig. 3I). These results provided compelling evidence to our notion that lactic acidosis in skeletal muscle causes age-dependent decreases in muscle force by downregulating *Ckmt2*, *Perm1*, and mitochondrially-encoded genes, contributing to the pathogenesis of sarcopenia.

We also analyzed the NADH/NAD^+^ ratio in skeletal muscle from 3–4-month-old, 18–20-month-old and 26–28-month-old mice, because the lactate/pyruvate ratio and the NADH/NAD^+^ ratio generally change in parallel (*30*). We observed decreases in NAD^+^ and NADH during aging (fig. S3, I and J). However, the NADH/NAD^+^ ratio remained unchanged during aging (fig. S3K). A specific condition exists in which the lactate/pyruvate ratio increases while the NADH/NAD^+^ ratio remains unchanged, which is characteristic of an acidotic environment, as the lactate/pyruvate ratio is governed by both proton concentration and the NADH/NAD^+^ ratio (*30*, *31*). This result also supports the occurrence of lactic acidosis in skeletal muscle of aged mice.

### Decreased systemic lactate processing capacity during aging

**Fig. 4.**
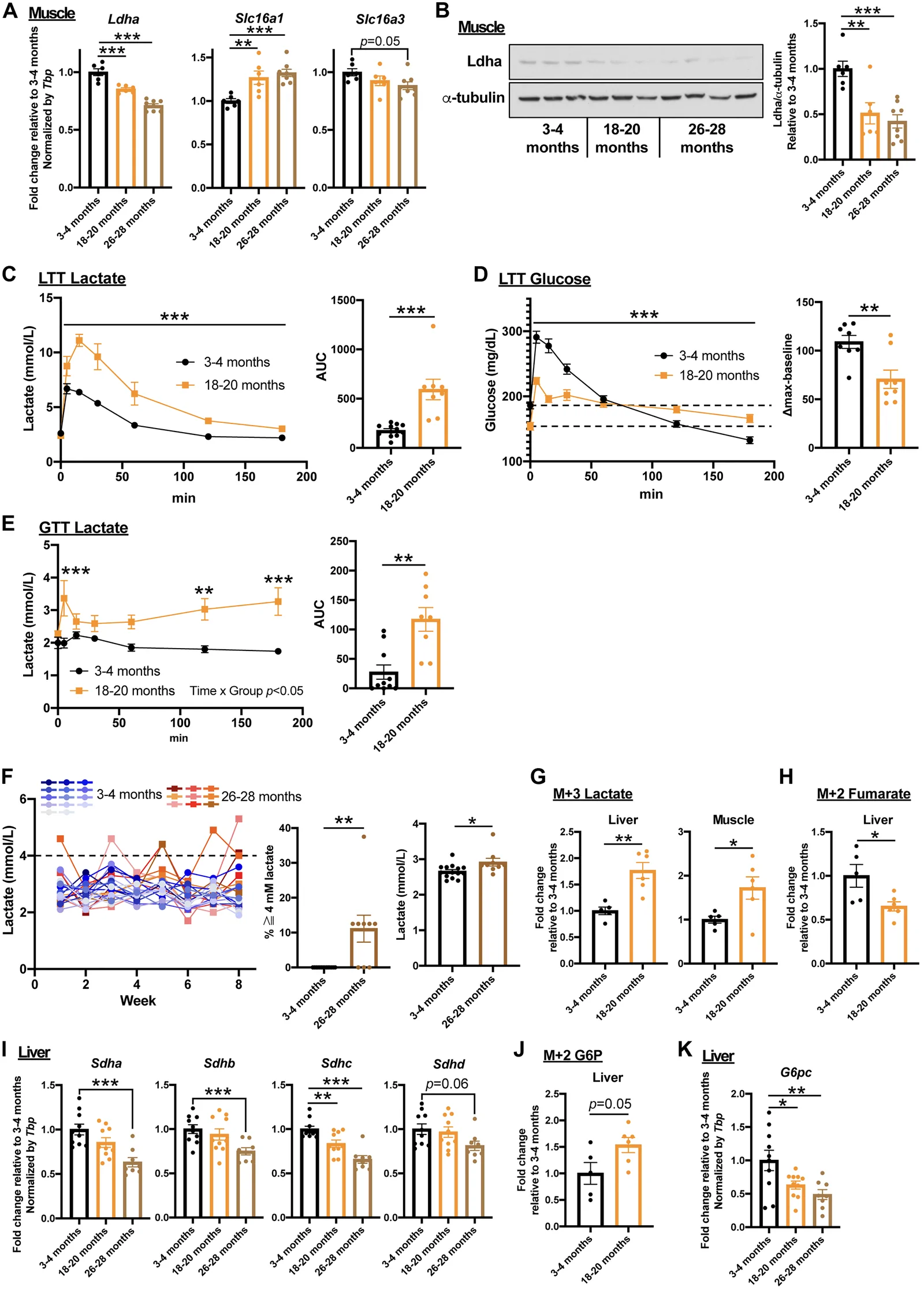
Decreased lactate processing capacity in the liver during aging. (**A**) mRNA levels of *Ldha*, *Slc16a1* and *Slc16a3* in the TA muscle of 3–4-month-old young, 18–20-month-old middle-aged, and 26–28-month-old aged mice. *n* = 6–8. (**B**) Representative western blotting of Ldha in the TA muscle of young, 18–20-month-old, and 26–28-month-old mice (left), and its quantification (right). *n* = 6–8. (**C**) Left: lactate tolerance test (LTT) in young or 18–20-month-old mice. Right: quantification of area under the curve (AUC) of LTT. *n* = 8–12. (**D**) Left: blood glucose levels during LTT. Right: quantification of differences between maximum and baseline glucose levels during LTT. *n* = 8. (**E**) Left: blood lactate levels during glucose tolerance test (GTT). Right: quantification of AUC of blood lactate levels during GTT. *n* = 8–10. (**F**) Left: weekly measurement of blood lactate levels in young or 26–28-month-old mice. Middle: quantification of the occurrence of high lactate levels (≧ 4 mM). Right: quantification of mean blood lactate levels for 2 months. *n* = 9–14. (**G**) Amount of ^13^C-lactate (M + 3) in the liver or muscle of young or 18–20-month-old mice after administration of ^13^C-lactate (M + 3). *n* = 5–6. (**H**) Amount of ^13^C-fumarate (M + 2) in the liver after administration of ^13^C-lactate. *n* = 5–6. (**I**) mRNA levels of *Sdha*, *Sdhb*, *Sdhc,* and *Sdhd* in the liver of young, 18–20-month-old, and 26–28-month-old mice. *n* = 8–10. (**J**) Amount of ^13^C-G6P (M + 2) in the liver after administration of ^13^C-lactate. *n* = 5–6. (**K**) mRNA levels of *G6pc* in the liver of young, 18–20-month-old, and 26–28-month-old mice. *n* = 8–10. *P < 0.05, **P < 0.01 and ***P < 0.001 by Student’s t-test for (C, right), (D, right), (E, right), (F, middle and right), (G), (H) and (**J**), by one-way ANOVA with Dunnett’s test for (A), (B), (I) and (K), or by two-way repeated-measures ANOVA with or without Sidak’s test for (C, left), (D, left) and (E, left). Error bars indicate s.e.m.

We next explored the cause of lactic acidosis in skeletal muscle of aged mice. We first hypothesized that increased production of lactate in skeletal muscle was the cause of lactic acidosis in skeletal muscle, and thus analyzed the expression of Ldha. Ldha is a major enzyme that produces lactate from pyruvate in skeletal muscle (*27*). Contrary to expectations, both gene expression and protein amounts of Ldha gradually decreased during aging in skeletal muscle (Fig. 4, A and B). A similar decrease in *LDHA* in human skeletal muscle during aging was reported (*32*). Given the gradual reduction in pyruvate in skeletal muscle during aging (Fig. 3B), these results indicated that both the precursor metabolite and the enzyme required to produce lactate were decreased during aging in skeletal muscle, suggesting that overproduction of lactate in skeletal muscle was *not* the cause of lactic acidosis in skeletal muscle. We therefore analyzed the expression of other lactate-related molecules. We observed an increased expression of *Slc16a1* [the gene encoding monocarboxylate transporter 1 (MCT1)] during aging in skeletal muscle (Fig. 4A). MCT1 is a transporter that imports extracellular lactate coupled with a proton (*33*, *34*). In contrast, the expression of *Slc16a3*, the gene encoding MCT4, which exports lactate out of the cell, tended to decrease during aging (Fig. 4A). Based on these results, we hypothesized that the increased lactate in skeletal muscle of aged mice did not result from the overproduction of lactate in skeletal muscle but from increased lactate uptake, and that muscle-extrinsic mechanisms were underlying lactic acidosis in skeletal muscle.

We therefore focused on the fundamental lactate metabolic pathway between the liver and skeletal muscle, the Cori cycle (*35*). The Cori cycle is a well-known metabolic pathway in which lactate, derived from skeletal muscle, is transported to the liver and converted into glucose by gluconeogenesis. This glucose is then released back into the bloodstream to fuel skeletal muscle (fig. S4A). The blood lactate level is strictly regulated by the liver and skeletal muscle (*36*). We hypothesized that a decline in lactate metabolism in the liver during aging caused reduced systemic lactate processing capacity, causing the accumulation of lactate in the blood. It is likely that skeletal muscle accepts the excess lactate, which contributes to lactic acidosis in skeletal muscle, because MCT1 transports extracellular lactate coupled with proton (fig. S4A) (*33*, *34*).

To address this hypothesis, we performed a lactate tolerance test (LTT) to analyze the changes in systemic lactate processing capacity during aging. Similar to a previous report from another group (*37*), an increase in blood lactate levels was observed after LTT in young mice (Fig. 4C). Eighteen-to-twenty-month-old mice showed much higher increases in blood lactate levels than young mice, indicating decreased lactate tolerance (Fig. 4C). A similar decrease in lactate tolerance was observed in 18–20-month-old female mice (fig. S4B). Because blood lactate in taken up by the liver and converted to glucose by gluconeogenesis, we analyzed the blood glucose levels during LTT. Young mice showed increased blood glucose levels immediately after the administration of lactate (Fig. 4D). Eighteen-to-twenty-month-old mice also showed increased blood glucose levels. However, the degree of increase in blood glucose levels was much lower in 18–20-month-old mice than in young mice (Fig. 4D), suggesting that the impaired lactate tolerance in 18–20-month-old mice resulted from decreased gluconeogenesis. Because lactate is produced by glycolysis, we analyzed blood lactate levels during a glucose tolerance test (GTT). Young mice showed mild increases in blood lactate levels after the administration of glucose (Fig. 4E). In contrast, 18–20-month-old mice showed a transient increase in blood lactate levels immediately after the administration of glucose (Fig. 4E). This transient increase in blood lactate levels subsided; however, 18–20-month-old mice exhibited a subsequent increase in blood lactate levels that was not observed in young mice (Fig. 4E), suggesting the prolonged increases in blood lactate levels after increased blood glucose levels, such as feeding, during aging. We therefore measured blood lactate levels once a week for 2 months. Twenty-six-twenty-eight-month-old mice did not exhibit chronic increases in blood lactate levels (Fig. 4F). However, 26–28-month-old mice showed higher blood lactate levels (≧4 mM) more frequently, and their frequency was significantly higher than that of young mice (Fig. 4F). The average blood lactate levels for 2 months were also higher in 26–28-month-old mice (Fig. 4F). These results suggest that, whereas each increase in blood lactate levels is transient and moderate, chronic increases in blood lactate levels could occur for a longer period of time during aging.

### Impaired lactate processing capacity in the liver during aging

To investigate the causes of impaired systemic lactate tolerance during aging, we administered stable isotope-labeled ^13^C-lactate (M + 3) to young and 18–20-month-old mice and analyzed ^13^C-lactate-derived metabolites and related genes (fig. S4C). Compared to vehicle-administered mice, we detected apparently higher signals of ^13^C-lactate (M + 3) both in the liver and skeletal muscle of ^13^C-lactate-administered mice (fig. S4D). Eighteen-to-twenty-month-old mice showed much higher increases in ^13^C-lactate (M + 3) both in the liver and skeletal muscle compared with young mice, indicating the increased uptake of lactate in skeletal muscle of 18–20-month-old mice (Fig. 4G), which is consistent with the increased expression of *Slc16a1* in skeletal muscle of 18–20-month-old mice (Fig. 4A). This result also indicated that impaired systemic lactate tolerance did not result from the inadequate lactate uptake in the liver (Fig. 4G). Given the impaired lactate tolerance in 18–20-month-old mice, the increase in ^13^C-lactate in the liver of 18–20-month-old mice suggested the impaired lactate processing capacity in the liver. The incorporated lactate is converted to pyruvate, which is then processed by the mitochondrial TCA cycle (fig. S4C) (*26*, *34*, *38*, *39*). The amount of ^13^C-pyruvate in the liver of 18–20-month-old mice was equivalent to that of young mice (fig. S4E), consistent with no change in *Ldha* expression in the liver during aging (fig. S4F). However, we found that the amount of ^13^C-fumarate (M + 2) was decreased in 18–20-month-old mice compared with that in young mice (Fig. 4H). There were no changes in the amount of ^13^C-citrate (M + 2) (fig. S4G). The amount of ^13^C-malate (M + 2) showed a slight decline but did not reach statistical significance (fig. S4G). We did not detect other M + 2 TCA cycle-related metabolites. These results suggest that the differences between young and 18–20-month-old mice become more pronounced as pyruvate is further metabolized. Furthermore, despite the high amount of ^13^C-lactate, the levels of its downstream metabolites were reduced in the liver of 18–20-month-old mice. We therefore analyzed the changes in TCA cycle-related gene expression in the liver during aging. Similar to a previous report from other groups (*40*), the genes related to the TCA cycle were globally downregulated during aging in the liver (Fig. 4I and fig. S4H). Although relatively milder than in male mice, similar decreases in the expression of *Sdha*, *Sdhd*, *Pdha*, and *Sucla2* were observed in the liver of female mice during aging (fig. S4I).

To analyze the contribution to gluconeogenesis, we analyzed the levels of ^13^C-lactate-derived glucose 6-phosphate (G6P) and fructose 6-phosphate (F6P). The levels of ^13^C-G6P tended to increase in the liver of 18–20-month-old mice (Fig. 4J), although the increase in ^13^C-F6P was not statistically significant (fig. S4J). The expression of *G6pc*, a rate-limiting enzyme for gluconeogenesis, was decreased in the liver of 18–20-month-old mice (Fig. 4K), suggesting that the reduced ability to generate glucose from G6P also contributed to the reduced lactate processing capacity in the liver during aging. This is consistent with dampened increases in blood glucose after the administration of lactate in 18–20-month-old mice (Fig. 4D). Based on these metabolic flux analyses, impaired systemic lactate tolerance during aging most likely results from decreased lactate processing capacity in the liver.

### PHD inhibitor alleviates lactic acidosis in skeletal muscle and age-related decline in muscle force

To further demonstrate the causal role of decreased lactate processing capacity in the liver for lactic acidosis in skeletal muscle, we examined whether amelioration of age-related declines in hepatic lactate metabolism alleviates lactic acidosis in skeletal muscle and sarcopenia. For this purpose, we focused on hypoxia-inducible factor (HIF) in the liver. It has been reported that the activation of HIF in the liver leads to increased lactate tolerance and alleviates systemic lactic acidosis by activating the Cori cycle (*41*, *42*). The activation of HIF can be achieved by inhibiting prolyl hydroxylase (PHD), which prevents the degradation of HIF by ubiquitin-proteasome under normoxia. Interestingly, PHD inhibitors are marketed as drugs for renal anemia (*43*). We therefore investigated the effects of a PHD inhibitor on lactic acidosis in skeletal muscle and sarcopenia.

Consistent with previous reports, treatment with roxadustat, a PHD inhibitor, resulted in the upregulation of HIF1α protein and *LDHA* and *SLC16A1* gene expression in HepG2 cells (fig. S5, A and B). Furthermore, a single intraperitoneal injection of roxadustat enhanced the expression of *Ldha* and *Slc16a1* in the liver of young mice (fig. S5C). We treated young mice with roxadustat three times per week for three weeks. These mice showed enhanced lactate tolerance in a dose-dependent manner (fig. S5D).

**Fig. 5.**
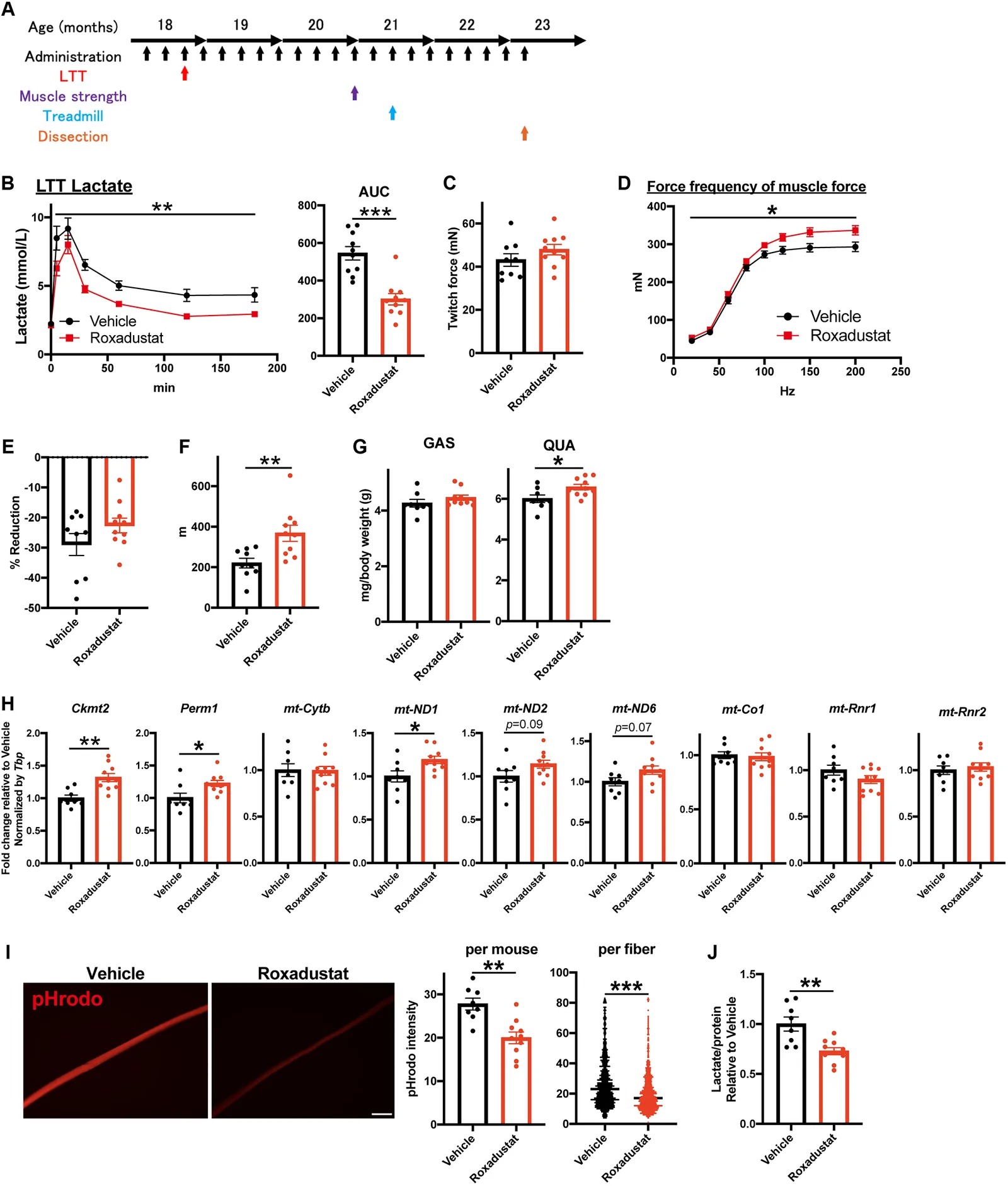
PHD inhibitor alleviates age-related decline in muscle force and lactic acidosis in skeletal muscle. (**A**) Schematic diagram of experimental design. (**B**) Left: lactate tolerance test (LTT) in vehicle- or roxadustat-administered mice. Right: quantification of area under the curve (AUC) of LTT in roxadustat-administered mice. *n* = 10. (**C**) Twitch force measurement in roxadustat-administered mice. *n* = 9–10. (**D**) In vivo muscle force-frequency curve in roxadustat-administered mice. *n* = 9–10. (**E**) Force reduction during repeated electrical stimulation in roxadustat-administered mice. *n* = 9–10. (**F**) Endurance capacity of roxadustat-administered mice by treadmill analysis. *n* = 9–10. (**G**) The GAS and QUA muscle weights of roxadustat-administered mice. *n* = 8–10. (**H**) mRNA expression levels of *Ckmt2*, *Perm1,* and mitochondrially-encoded genes in roxadustat-administered mice. *n* = 8–10. (**I**) Left: representative fluorescence image of pHrodo in single muscle fibers from vehicle- or roxadustat-administered mice. Bar: 100 μm. Right: quantitative analysis of pHrodo intensity in single muscle fibers from roxadustat-administered mice per mouse (left) and per fiber (right). *n* = 8–10. (**J**) Amounts of lactate in the TA muscle of roxadustat-administered mice. *n* = 8–10. *P < 0.05, **P < 0.01 and ***P < 0.001 by Student’s t-test for (B, right), (F), (G), (H), (I) and (J), or by two-way repeated-measures ANOVA for (B, left) and (D). Error bars indicate s.e.m.

We then examined the effects of roxadustat on lactic acidosis in skeletal muscle and sarcopenia. We administered roxadustat to 18-month-old mice intraperitoneally three times per week (Fig. 5A). As expected, roxadustat-treated mice showed enhanced systemic lactate tolerance compared with the vehicle-treated control mice (Fig. 5B). Remarkably, the age-related decrease in muscle force was significantly alleviated by roxadustat, although the difference in twitch force and fatigue resistance did not reach statistical significance (Fig. 5, C to E). Specific force was also significantly alleviated by roxadustat, although 100% contraction time and 50% relaxation time were not altered (fig. S5, E to G). Furthermore, endurance capacity, as measured by treadmill running, was also enhanced by roxadustat (Fig. 5F). We next measured muscle weight. Roxadustat-treated mice showed increased quadriceps (QUA) muscle weight, but did not show changes in the weights of other hindlimb muscles such as the GAS, TA, EDL, soleus (SOL), or plantaris (PLA) muscle (Fig. 5G and fig. S5H). Consistent with the increased muscle force, the expression levels of *Ckmt2*, *Perm1*, and mitochondrially-encoded genes were upregulated in roxadustat-treated mice (Fig. 5H). To examine the effects of roxadustat on age-related lactic acidosis in skeletal muscle, we analyzed pHrodo intensity and lactate levels. The pHrodo intensity was decreased in myofibers from roxadustat-treated mice (Fig. 5I). Lactate levels in skeletal muscle were also reduced in roxadustat-treated mice (Fig. 5J). We did not observe any changes in the number of Fluo-4 abnormal fibers by following roxadustat treatment (fig. S5I). These results clearly demonstrate that roxadustat is able to alleviate age-related decreases in systemic lactate tolerance, lactic acidosis in skeletal muscle, and age-related decline in muscle force.

### Liver-specific overexpression of constitutively active *HIF1*α alleviates lactic acidosis in skeletal muscle and age-related decline in muscle force

Because the PHD inhibitor acts systemically, we specifically activated HIF in the liver by injecting an adeno-associated virus (AAV) expressing constitutively active *HIF1a* (*c.a.HIF1*α) under the control of the liver-specific human thyroxine-binding globulin (TBG) promoter (AAV-TBG-*c.a.HIF1*α). *Enhanced green fluorescence protein* (*EGFP*) was used as a control (AAV-TBG-*EGFP*). We first injected AAV-TBG-*c.a.HIF1*α or -*EGFP* into young mice via the tail vein. AAV-TBG-*c.a.HIF1*α-treated young mice showed increased HIF1α protein and upregulation of *Ldha* and *Slc16a1* in the liver (fig. S6, A and B), which is similar to the effects of roxadustat administration (fig. S5, A and C). AAV-TBG-*c.a.HIF1*α-treated young mice also showed enhanced systemic lactate tolerance (fig. S6C).

**Fig. 6.**
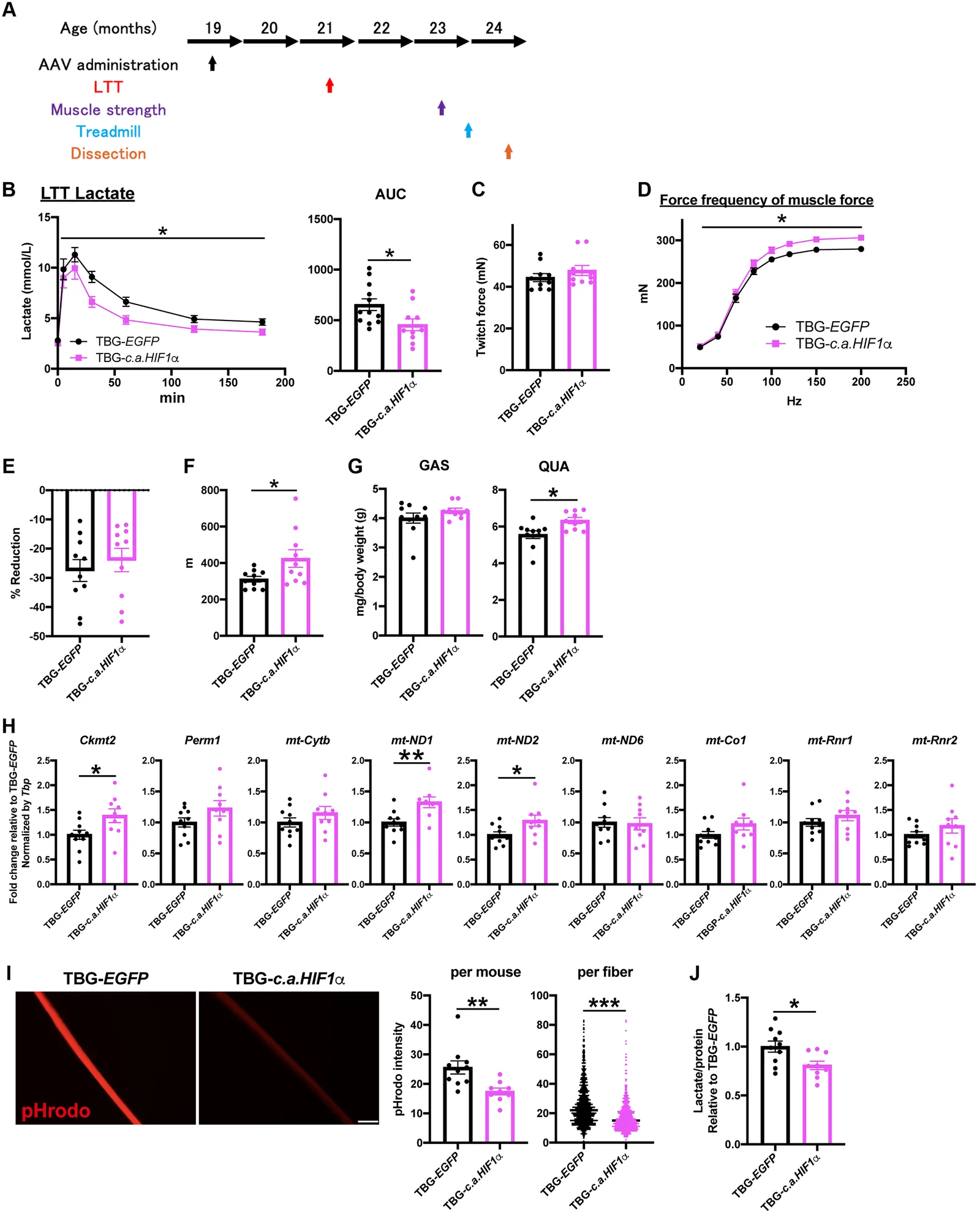
Liver-specific overexpression of constitutively active *HIF1*α alleviates age-related decline in muscle force and lactic acidosis in skeletal muscle. (**A**) Schematic diagram of experimental design. (**B**) Left: lactate tolerance test (LTT) in *EGFP*- or *c.a.HIF1*α-overexpressed mice. Right: quantification of area under the curve (AUC) of LTT in *c.a.HIF1*α-overexpressed mice. *n* = 10–12. (**C**) Twitch force measurement in *c.a.HIF1*α-overexpressed mice. *n* = 10. (**D**) In vivo muscle force-frequency curve in *c.a.HIF1*α-overexpressed mice. *n* = 10. (**E**) Force reduction during repeated electrical stimulation in *c.a.HIF1*α-overexpressed mice. *n* = 10. (**F**) Endurance capacity of *c.a.HIF1*α-overexpressed mice by treadmill analysis. *n* = 10. (**G**) The GAS and QUA muscle weights of *c.a.HIF1*α-overexpressed mice. *n* = 9–10. (**H**) mRNA expression levels of *Ckmt2*, *Perm1* and mitochondrially-encoded genes in *c.a.HIF1*α-overexpressed mice. *n* = 9–10. (**I**) Left: representative fluorescence image of pHrodo in single muscle fibers from *EGFP*- or *c.a.HIF1*α-overexpressed mice. Bar: 100 μm. Right: quantitative analysis of pHrodo intensity in single muscle fibers from *c.a.HIF1*α-overexpressed mice per mouse (left) and per fiber (right). *n* = 9–10. (**J**) Amounts of lactate in the TA muscle *c.a.HIF1*α-overexpressed mice. *n* = 9–10. *P < 0.05, **P < 0.01 and ***P < 0.001 by Student’s t-test for (B, right), (F), (G), (H), (I) and (J), or by two-way repeated-measures ANOVA for (B, left) and (D). Error bars indicate s.e.m.

We then treated 19-month-old mice with AAV-TBG-*c.a.HIF1*α or –*EGFP* (Fig. 6A). Similar to the effects observed in roxadustat-treated mice, AAV-TBG-*c.a.HIF1*α significantly enhanced systemic lactate tolerance, muscle force, and endurance capacity (Fig. 6, B to F). Specific force was also significantly enhanced by AAV-TBG-*c.a.HIF1*α, although 100% contraction time and 50% relaxation time were not changed (fig. S6, D to F). Furthermore, AAV-TBG-*c.a.HIF1*α significantly increased the weight of the QUA muscle, but not of other hindlimb muscles (Fig. 6G and fig. S6G). AAV-TBG-*c.a.HIF1*α-treated mice also showed enhanced *Ckmt2*, *mt-ND1*, and *mt-ND2* expression in skeletal muscle (Fig. 6H). Decreased pHrodo intensity and lactate levels in skeletal muscle were also observed in AAV-TBG-*c.a.HIF1*α-treated mice (Fig. 6, I and J). No changes were observed in the number of Fluo-4 abnormal fibers following AAV-TBG-*c.a.HIF1*α treatment (fig. S6H). These results indicate that the activation of HIF in the liver enhances systemic lactate tolerance and alleviates lactic acidosis in skeletal muscle and age-related decline in muscle force, suggesting that liver HIF is a druggable target for age-associated sarcopenia.

### Skeletal muscle NAD^+^ as an intermediary of lactic acidosis in skeletal muscle and age-related decline in muscle force

**Fig. 7.**
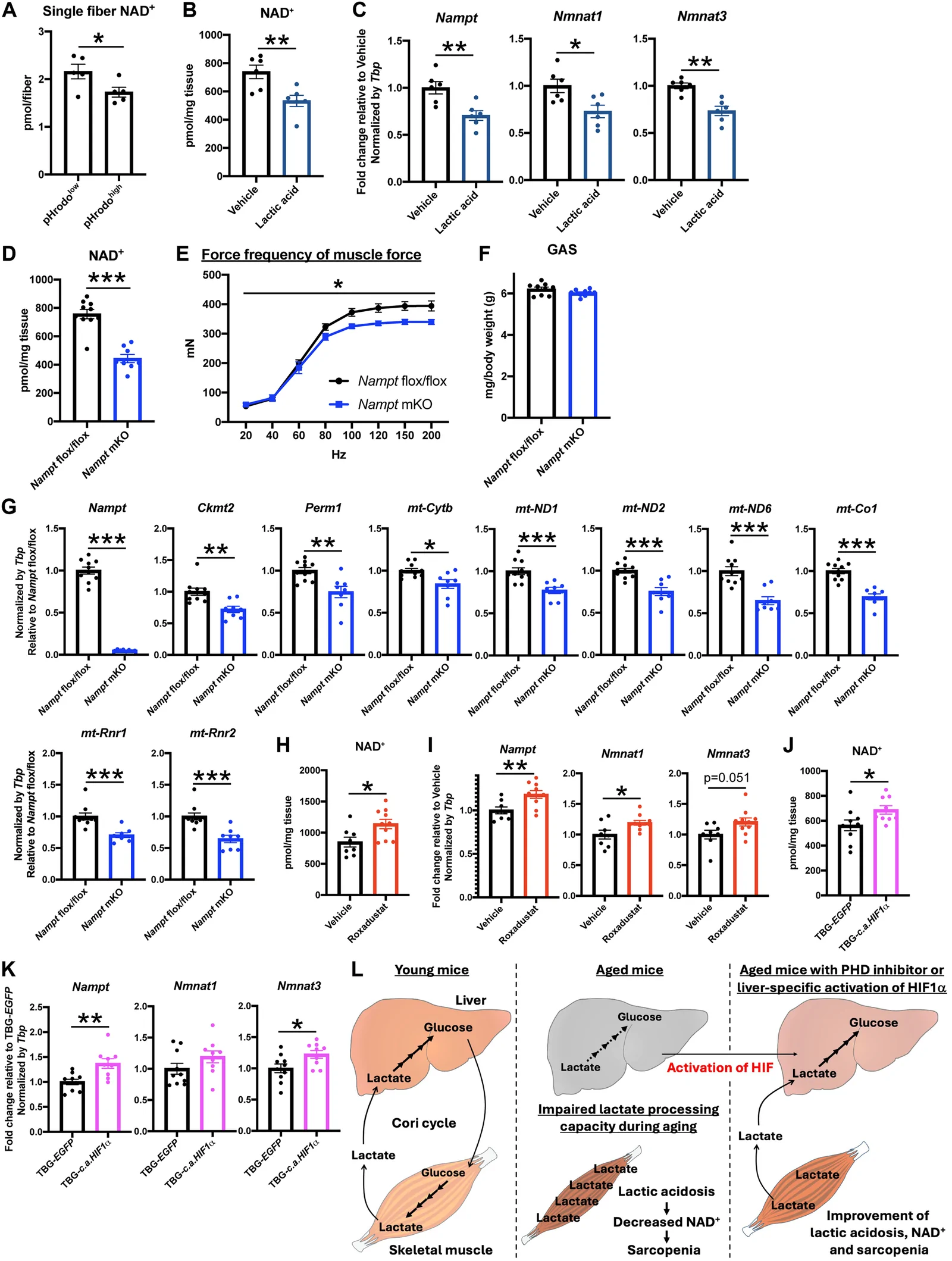
Skeletal muscle NAD^+^ as an intermediary of lactic acidosis and age-related decline in muscle force. (**A**) NAD^+^ levels in pHrodo^low^ or pHrodo^high^ single muscle fibers from 26–28-month-old aged mice. *n* = 5. (**B**) NAD^+^ levels in the GAS muscles of lactic acid-administered mice. *n* = 6. (**C**) mRNA expression levels of *Nampt*, *Nmnat1* and *Nmnat3* in the GAS muscles of lactic acid-administered mice. *n* = 6. (**D**) NAD^+^ levels in the TA muscles of *Nampt* mKO mice. *n* = 8–10. (**E**) In vivo muscle force-frequency curve in *Nampt* mKO mice. *n* = 6–7. (**F**) The GAS muscle weights in *Nampt* mKO mice. *n* = 9. (**G**) mRNA expression levels of *Nampt*, *Ckmt2*, *Perm1,* and mitochondrially-encoded genes in *Nampt* mKO mice. *n* = 8–10. (**H**) NAD^+^ levels in the TA muscles of roxadustat-administered mice. *n* = 8–10. (**I**) mRNA expression levels of *Nampt*, *Nmnat1* and *Nmnat3* in the TA muscles of roxadustat-administered mice. *n* = 8–10. (**J**) NAD^+^ levels in the TA muscles of liver-specific *c.a.HIF1*α-overexpressed mice. *n* = 9–10. (**K**) mRNA expression levels of *Nampt*, *Nmnat1* and *Nmnat3* in the TA muscles of liver-specific *c.a.HIF1*α-overexpressed mice. *n* = 9–10. (**L**) Scheme for lactic acidosis in skeletal muscle owing to impaired liver-skeletal muscle lactate metabolism during aging, and the effects of PHD inhibitor or liver-specific activation of HIF on sarcopenia. *P < 0.05, **P < 0.01 and ***P < 0.001 by Student’s t-test for (A), (B), (C), (D), (G), (H), (I), (J) and (K), or by two-way repeated-measures ANOVA for (E). Error bars indicate s.e.m.

We investigated an essential intermediary that possibly connects lactic acidosis in skeletal muscle, decreased muscle force, and key gene expression changes. Migliavacca *et al.* (*7*). demonstrated that skeletal muscle NAD^+^ levels were decreased in older adults with sarcopenia. Our gene-disease network analysis based on their transcriptomic data (fig. S3A), indicated that both lactic acidosis in skeletal muscle and decreases in NAD^+^ levels occurred in older adults with sarcopenia. We therefore examined the cause-and-effect relationship between lactic acidosis in skeletal muscle and skeletal muscle NAD^+^ levels. We isolated pHrodo^low^ and pHrodo^high^ single muscle fibers from 26–28-month-old mice and analyzed the levels of NAD^+^. Interestingly, we observed decreased NAD^+^ levels in pHrodo^high^ fibers (Fig. 7A). Additionally, intramuscular injection of lactic acid in young mice caused a reduction in NAD^+^ levels (Fig. 7B). *Nicotinamide phosphoribosyltransferase* (*Nampt*), a rate-limiting enzyme for the NAD^+^ salvage pathway, *Nicotinamide mononucleotide adenylyltransferase 1 (Nmnat1)* and *Nmnat3* were downregulated by the injection of lactic acid, suggesting a decrease in NAD^+^ synthesis by lactic acidosis (Fig. 7C).

To further examine the role of skeletal muscle NAD^+^ in muscle force, we established skeletal muscle-specific *Nampt* knockout mice. We crossed *Nampt*^flox/flox^ mice with mice expressing the inducible mutated estrogen receptor fused Cre recombinase (MerCreMer) under the control of the human skeletal muscle actin (HSA) promoter (HSA-MCM) (*44*, *45*). To analyze the effects of decreased NAD^+^ levels, specifically in adults, we administered tamoxifen to 3–4-month-old mice. One week after the administration of tamoxifen, *Nampt* muscle knockout mice (*Nampt* mKO) showed a significant reduction in skeletal muscle NAD^+^ levels by approximately 50% (Fig. 7D). At the same time, *Nampt* mKO mice showed decreased muscle force but not muscle weight (Fig. 7, E and F), indicating the requirement of skeletal muscle NAD^+^ in the maintenance of muscle force. We also observed a decline in specific force, but no changes in 100% contraction time or 50% relaxation time (fig. S7, A to C). In addition to the reduction of *Nampt* expression in *Nampt* mKO mice, the expression levels of *Ckmt2*, *Perm1*, and mitochondrially-encoded genes were also decreased in *Nampt* mKO mice (Fig. 7G). In addition to the in vivo knockout of *Nampt*, we knocked down *Nampt* in primary myotubes by shRNA. Knockdown of *Nampt* caused a reduction in NAD^+^, and its knockdown efficiency was approximately 98% (fig. S7, D and E). Knockdown of *Nampt* in vitro resulted in the reduction of *Perm1* and mitochondrially-encoded genes, although there was no change in the expression of *Ckmt2* (fig. S7E). Knockdown of *Sirtuin 1* (*Sirt1*) also caused similar decreases in *Perm1*, *mt-Cytb*, *mt-ND1*, *mt-ND2*, and *mt-Rnr2* (fig. S7F). These results suggest that skeletal muscle NAD^+^ and its downstream *Sirt1* are required to maintain muscle force by regulating the *Ckmt2*/*Perm1*-mitochondrially-encoded gene axis.

Finally, we examined the effects of the PHD inhibitor or liver-specific activation of HIF1α on skeletal muscle NAD^+^ levels in 18–19-month-old mice. The administration of roxadustat or liver-specific activation of HIF1α resulted in increased NAD^+^ levels in skeletal muscle, which was accompanied by increased expression of *Nampt* (Fig. 7, H to K). We also observed the increased expression of *Nmnat1* by roxadustat and *Nmnat3* by liver-specific activation of HIF1α (Fig. 7, I and K). Taken together, these results firmly demonstrate that the age-related dysfunction of lactate metabolism in the liver causes lactic acidosis in skeletal muscle, which decreases skeletal muscle NAD^+^ levels and the downstream *Ckmt2*/*Perm1*-mitochondirally encoded gene axis, causally contributing to the pathogenesis of sarcopenia during aging (Fig. 7L).

## DISCUSSION

The Cori cycle is a key lactate metabolic pathway between the liver and skeletal muscle. Our data indicated that impaired Cori cycle induced lactate accumulation in skeletal muscle during aging, which led to muscle dysfunction. Our stable isotope experiments suggested that enhanced uptake of lactate by skeletal muscle was a compensatory mechanism for impaired lactate metabolism in the liver, because lactate processing capacity was decreased in the liver of 18–20-month-old mice (Fig. 4 and fig. S4). In addition, our gene-disease network analysis suggested the involvement of lactic acidosis in human sarcopenic muscle (fig. S3A) (*7*). A recent multi-omics study on sarcopenia included lactate among the list of metabolites elevated in older adults with sarcopenia compared with healthy controls (*46*). These findings strengthen the importance of lactic acidosis in skeletal muscle as a fundamental cause of sarcopenia. Conversely, a recent human plasma metabolomics study reported that blood lactate levels were not elevated in older adults with sarcopenia (*47*). Given that lactate concentrations are highly sensitive to prior exercise and dietary intake, more strictly controlled study designs are required. It is also possible that a LTT is necessary to reveal underlying metabolic impairment in older adults with sarcopenia, analogous to the use of a GTT to detect impaired glucose metabolism. This interpretation is further supported by findings in septic patients, in whom sarcopenia was associated with significantly higher blood lactate levels (*48*). Collectively, these findings suggest that differences in blood lactate levels may only emerge when the lactate-buffering system is physiologically stressed. Further studies will be required to verify the contribution of lactic acidosis in skeletal muscle to the pathogenesis of sarcopenia in human.

Mitochondria have a key role in the maintenance of muscle force. Indeed, mitochondrial function in skeletal muscle is compromised in older adults with sarcopenia (*7*). Our data indicated that extrinsic lactate reduces muscle force by downregulating *Ckmt2*, *Perm1*, and mitochondrially-encoded genes (Fig. 3, G to I), suggesting that muscle lactic acidosis impairs mitochondrial function. Fast muscle is significantly impaired in sarcopenia compared with slow muscle, possibly because of differences in the ability to process lactate. Mitochondria-enriched slow muscle takes up lactate and uses it as an energy source (*33*). Fast muscle has fewer mitochondria and, by its nature, is less adept at processing lactate than slow muscle. Our results suggested that the uptake of lactate by fast muscles, which are not specialized in lactate processing, and subsequent dysfunction of mitochondria are causes of fast muscle dysfunctions in sarcopenia.

In sarcopenia, loss of muscle force precedes loss of muscle mass (*49*). Furthermore, a previous report highlights muscle force, but not muscle mass, as an indicator of mortality risk (*6*). Our results indicated that lactic acidosis in skeletal muscle primarily affects muscle force because a single intramuscular injection of lactic acid led to decreased muscle force, but not muscle mass (Fig. 3, G and H and fig. S3E). This is consistent with no changes in muscle weight after knockdown of *Cktm2* or *Perm1*, or knockout of *Nampt* (Figs. 2E and 7F). Given the apparent decrease in systemic lactate tolerance in 18–20-month-old mice (Fig. 4C), lactic acidosis in skeletal muscle may be involved in the relatively early pathogenesis of sarcopenia. Notably, it has been reported that long-term systemic exposure to lactate induces loss of muscle mass (*50*). Prolonged lactic acidosis in skeletal muscle, combined with the influence of other negative factors such as systemic inflammation and malnutrition, may lead to decreases in muscle mass.

Although our data indicated the essential role of lactic acidosis in skeletal muscle in the pathogenesis of sarcopenia, several studies have revealed the positive effects of lactate for the maintenance of muscle homeostasis (*26*). This reciprocal effect likely results from the difference between lactate and lactic acid. Systemic energy expenditure and carbohydrate expenditure decrease during aging (*29*). The amount of pyruvate, an end product of glycolysis, and expression of *Ldha*, which produces lactate from pyruvate, were also decreased during aging in skeletal muscle (Figs. 3B and 4, A and B) (*29*, *32*), suggesting that intrinsic lactate production is reduced during aging. Nevertheless, our data indicated a gradual increase in lactate in skeletal muscle during aging (Fig. 3C). Of note, MCT1, a transporter encoded by *Slc16a1*, takes up lactate coupled with proton (*34*). Given the upregulation of *Slc16a1* in aged muscle (Fig. 4A) and increased uptake of stable isotope-labeled lactate in skeletal muscle (Fig. 4G), the increased uptake of extrinsic lactate and proton (lactic acid) leads to accumulation of lactate and lactic acidosis in skeletal muscle during aging. The excessive increases in the lactate/pyruvate ratio in skeletal muscle during aging likely reflect the excessive uptake of extrinsic lactate (Fig. 3D). In addition to increased lactate in skeletal muscle, we demonstrated frequent increases in blood lactate levels in aged mice (Fig. 4F). Of note, the elevation of blood lactate levels was also observed in other muscle-wasting disorders, such as cachexia and amyotrophic lateral sclerosis (*50*, *51*), suggesting that impaired lactate metabolism may contribute to the pathogenesis of other muscular diseases.

Our results demonstrate the importance of lactic acidosis in skeletal muscle. However, we could not clarify the relationship between sarcopenia and other metabolic acidosis, such as ketoacidosis. Association between dietary acid load and the prevalence of sarcopenia, or that between decreased urinary pH and sarcopenia, has been reported (*52*, *53*), suggesting that acidosis, not limited to lactic acidosis, may be involved in sarcopenia. Future research focusing on diet and nutrients that prevent metabolic acidosis is expected.

Our data demonstrated that the pharmacological and genetic activation of HIF enhanced systemic lactate tolerance and improved age-related decline in muscle force (Figs. 5 and 6). PHD inhibitors are marketed as drugs for renal anemia (*43*). Thus, our study highlights liver HIF as a therapeutic target for sarcopenia and the potential therapeutic application of PHD inhibitors through drug repositioning. Although our study demonstrated the potential effectiveness of PHD inhibitors for sarcopenia, the contribution of intrinsic HIF to the pathogenesis of sarcopenia or age-associated decreases in lactate processing capacity was unclear. Treatment with roxadustat or liver-specific activation of HIF caused increased expression of *Ldha* (figs. S5C and S6B). However, there was no change in the expression of *Ldha* in the liver during aging (fig. S4F). Furthermore, a previous study from another group showed that age-associated activation of HIF1α in skeletal muscle contributed to mitochondrial abnormalities by negatively affecting Sirt1 (*12*). This previous study also reinforced that roxadustat ameliorated lactic acidosis in skeletal muscle and the age-related decline in muscle force by activating HIF in the liver, not skeletal muscle. Further studies are required to investigate the tissue-specific role of HIF in the pathogenesis of sarcopenia. It is possible that mitochondrial abnormalities in skeletal muscle trigger lactic acidosis, as lactate uptake was increased following the knockout of *mitofusin 1/2* in skeletal muscle (*54*). In the complex landscape of sarcopenia, both liver-derived and skeletal muscle-derived factors are likely to occur simultaneously, although their causal and reciprocal relationships remain to be elucidated in future studies.

Our results indicated that NAD^+^ is an essential mediator that connects lactic acidosis in skeletal muscle and sarcopenia (Fig. 7). Lactic acidosis in skeletal muscle induced downregulation of *Nampt*, *Nmnat1* and *Nmnat3* and subsequent decreases in NAD^+^ levels, which in turn caused the decreases in the *Ckmt2*/*Perm1*-mitochondrially-encoded genes axis. A similar decrease in *NAMPT* and *NMNAT1* was observed in older adults with sarcopenia (*7*), suggesting the critical role of NAD^+^ in the pathogenesis of sarcopenia. Our results showed that lactic acidosis in skeletal muscle caused a reduction in skeletal muscle NAD^+^ (Fig. 7B). The phenotype of *Nampt* mKO may recapitulate decreases in NAD^+^ owing to lactic acidosis in skeletal muscle during aging. A previous study from other group showed that acute lactate treatment did not impair excitation-contraction coupling (*55*). This is consistent with our data, which suggest that chronic lactic acidosis in skeletal muscle leads to progressive muscle dysfunction during aging through a reduction in NAD^+^ levels during aging. In contrast, Chubanava S *et al.* (*13*). recently showed that the depletion of NAD^+^ in skeletal muscle did not affect muscle function. We observed the decreases in muscle force 1 week after the knockout of *Nampt*, whereas Chubanava S *et al.* (*13*). analyzed *Nampt* mKO at approximately 2 months after the knockout of *Nampt*. These results imply that the phenotype of *Nampt* mKO changes in a time-dependent manner. The more precise time-course analysis is required to clarify the significance of NAD^+^ in skeletal muscle in the future.

There are several limitations in this study. We demonstrated that intraperitoneal administration of roxadustat enhanced lactate tolerance and muscle force in aged mice. The dosages used were much higher than those in clinical use (*56*). To investigate the effects of PHD inhibitors on sarcopenia, further studies will be needed to explore the optimal dose of roxadustat or other PHD inhibitors with consideration of side effects.

In addition, our study started with the identification of the Fluo-4 abnormal fibers in aged mice (Fig. 1). We found that intracellular acidification was more prominent in these abnormal fibers (Fig. 3, E and F), but we did not address the relationship between intracellular acidification and the formation of tubular aggregate-like structures. Although tubular aggregates occur specifically in male mice (*17*, *18*), intracellular acidification occurred in female mice during aging (fig. S3D). These results suggested that tubular aggregate is a marker of abnormal muscle fibers, but intracellular acidification does not promote the formation of tubular aggregate. There may not be a direct relationship between intracellular acidification and the formation of tubular aggregate. In addition, although we observed impaired Ca^2+^ handling in the aged Fluo-4 abnormal fibers, the cause-and-effect relationship between the development of tubular aggregate-like structures and altered Ca^2+^ handling remains to be elucidated. Furthermore, although we have demonstrated the significance of *Ckmt2* and *Perm1* through knockdown experiments (Fig. 2), their specific roles in aging-related pathology require further validation through overexpression studies. This objective will be addressed in future research.

Finally, lactate is also processed by the kidney. Although our liver-specific overexpression of *c.a.HIF1a* results indicated the importance of the liver for the pathogenesis of lactic acidosis in skeletal muscle, the contribution of the kidney could not be examined in this study. Because the kidney is composed of a wide variety of cells, and useful kidney-specific promoters for AAV are lacking, the relationship between renal lactate metabolism and sarcopenia should be investigated using transgenic and other mouse models in the future. Of note, the prevalence of sarcopenia is increased in patients with hepatic or renal insufficiency (*4*, *5*, *57*), and the abnormal lactate metabolism owing to hepatic and/or renal insufficiency is possibly related to sarcopenia.

In summary this study identified lactic acidosis in skeletal muscle as a fundamental molecular pathogenesis of sarcopenia. The impaired lactate metabolism between skeletal muscle and the liver during aging induced the accumulation of lactic acid in skeletal muscle. This lactic acidosis decreased skeletal muscle NAD^+^ levels and its downstream *Cktm2*/*Perm1*-mitochondrially-encoded genes axis, leading to reduced muscle force. Furthermore, age-related dysfunction of lactate metabolism and sarcopenia was rescued by pharmacological activation of HIF or liver-specific activation of HIF1α, indicating that liver HIF is a drug target for sarcopenia.

## MATERIALS AND METHODS

### Animals

C57BL/6 J mice were purchased from the Jackson Laboratory (Kanagawa, Japan). Aged mice were obtained from National Center for Geriatrics and Gerontology (NCGG) (Obu, Aichi, Japan). *Nampt* flox mice were a kind gift from Dr. Shin-ichiro Imai (*44*). *HSA-MerCreMer* mice were purchased from the Jackson Laboratory (*45*). Intraperitoneal injections of 100 mg/kg tamoxifen (Fujifilm Wako, QC-0156) in corn oil were performed to 3–4-month-old mice for 5 days. All mice were housed at the NCGG on a 12 h–12 h light/dark cycle with free access to water and standard mouse chow. All animal procedures were approved by the Experimental Animal Care and Use Committee of the NCGG (approval number: Animal7–29-R3). Mice at 3–4, 18–20, and 26–28 months of age were defined as young, middle-aged, and aged mice, respectively.

For roxadustat administration, a solution with 3 g/L roxadustat in 0.5% carboxymethylcellulose sodium and 0.1% polysorbate 80 was prepared as previously described (*58*). Ten or thirty mg/kg body weight of roxadustat was intraperitoneally injected.

For intramuscular injection of lactic acid, mice were anesthetized with isoflurane on a heated plate. L-lactic acid in saline (0.05%, 6 mM and approximately pH 3.0) was injected into the GAS muscle. These mice were analyzed 10 h or 1 day after lactic acid injection.

For Figs. 2C, 3 (B to D), 4 (A, B, and G to K), 5 (F to J), 6 (F to J), 7 (D and F to K), figs. S3 (I to K), S4 (D to J), S5 (H and I), and S6 (G to H), mice were analyzed or sacrificed around ZT13:00. Other mice were analyzed or sacrificed at daytime.

### Skeletal muscle functional test

In vivo muscle force was analyzed using 1300A (Aurora Scientific, Canada) as previously described with minor modifications (*29*). Mice were anesthetized with isoflurane on a heated plate. The hindlimb ankles were positioned at 90° flexion, and muscle force was measured using indirect electrical stimulation of the plantar flexor muscles. Twitch force was electrically evoked with a single 0.2 msec pulse. Tetanic force was measured at 100 Hz for a 0.35 s pulse in young mice and at 200 Hz in aged mice. For force-frequency curve analysis, 20, 40, 60, 80, 100, 120, 150, and 200 Hz for 0.35 s with 1 min interval was applied. A fatigue resistance test was performed with 60 repeated electrical stimulations at 2 s intervals of 30 Hz for 0.35 s. The reduction from maximum to minimum force was calculated as a percentage reduction. Time to peak contraction (100% contraction time) and half-relaxation time (50% relaxation time) were determined from twitch force recordings. Specific force was calculated by dividing tetanic force by the cross-sectional area of the GAS muscle.

The treadmill exercise experimental model was performed as described previously, with modifications using MK-680 (Muromachi Kikai) or TMS-6B (MELQUEST) (*29*). Briefly, mice were forced to run at 5 m/min for the first 5 min. The treadmill speed was increased by 1 m/min every minute for 20 min until it reached 25 m/min, after which the mice were forced to run at a constant speed until exhaustion.

### Immunohistochemical analysis

Immunohistochemical analyses were performed as described previously with minor modifications (*29*). Briefly, isolated GAS muscles were frozen in isopentane cooled in liquid nitrogen. Cryosections of 8 μm thickness were cut across the middle part of the GAS muscle. After air-drying, the sections were fixed with acetone for 10 min. Sections were blocked using 5% goat serum/1% bovine serum albumin (BSA) and phosphate buffered saline (PBS) for 15 min at room temperature. The sections were stained overnight with primary antibodies: anti-RyR (DSHB, 34C, 1:4,000), anti-DHPR (NOVUS, NB300–542, 1:4000), and anti-SERCA (GENETEX, GTX22819, 1:50,000) antibodies in 1% BSA/PBS at 4°C. After several washes with PBS, the sections were stained overnight with Alexa Fluor-conjugated secondary antibody in 1% BSA/PBS at 4°C. After washing and staining with 4′,6-diamidino-2-phenylindole (DAPI), the sections were mounted with Fluoromount.

### RNA isolation, reverse transcription-polymerase chain reaction analysis, and RNA-seq analysis

Isolation of total RNA and subsequent polymerase chain reaction (PCR) analysis were performed as described previously with modifications (*29*). Briefly, isolated tissues or cells were immediately frozen in liquid nitrogen. Total RNA was extracted using the TRIzol reagent (Invitrogen). Single-stranded cDNA synthesis was performed using Superscript IV VILO Master Mix (Invitrogen). For quantitative RT-PCR (qRT-PCR), the expression levels of the target genes were evaluated using TB Green Premix Ex Taq II (Takara Bio) on a CFX Opus96 (Bio-Rad). The primers used for qRT-PCR are listed in table S1. The expression level of each target gene was normalized to that of *Tata-box binding protein* (*Tbp*).

RNA-seq analysis was performed using Takara. Sequenced reads were aligned to the mouse reference genome (GRCm39/mm39) using DRAGEN (Ver. 4.3). Gene expression counts and transcripts per million were calculated using the DRAGEN software. The RNA-seq data were analyzed using the Subio platform. Annotation with “protein_coding” was extracted for all genes. Genes with low expression (value less than 0.02 in all groups) were excluded from the analysis. Genes with two-fold increase or decrease in expression compared with that in young single fibers were defined as differentially expressed genes. Among these, genes that were upregulated or downregulated in the Fluo-4 abnormal fibers compared with those in the Fluo-4 normal fibers (*P* value <0.05) were analyzed using Metascape. RNA-seq data were deposited in Gene Expression Omnibus under the accession number GSE300563. For previously published RNA-seq data from Migliavacca *et al.* (*7*)., genes with negative coefficient of association with sarcopenia and a *P* value <0.05 were extracted, and analyzed by Metascape (*24*) and DisGeNET (*25*).

### Western blotting

Western blotting was performed as described previously with minor modifications (*29*). Briefly, the TA muscle, liver, or cells were immediately frozen in liquid nitrogen. The TA muscles were dissected using a cryostat. Total proteins were extracted using a sample buffer containing 50 mM HEPES (pH 7.4), 100 mM glycerophosphate, 15 mM Na_4_P_2_O_7_, 4 mM EGTA, 10 mM EDTA, 5 mM Na_2_VO_4_, 0.1% Triton X-100, 25 mM NaF, and protease inhibitor cocktail (Roche, Basel, Switzerland). Protein concentration was determined using Coomassie Brilliant Blue G-250 (Bio-Rad, Hercules, CA, USA). The extracted protein solution was mixed with an equal volume of sample loading buffer containing 30% glycerol, 5% 2-mercaptoethanol, 2.3% SDS, 62.5 mM Tris-HCl (pH 6.8), and 0.05% bromophenol blue. The mixed solution was heated at 60°C for 10 min. The extracted proteins were separated on a sodium dodecyl sulfate-polyacrylamide gel and transferred onto a polyvinylidene difluoride membrane (Millipore, Burlington, MA, USA). The ECL western blotting detection system was used for signal detection. Antibodies against LDHA (CST, #2012, 1:20,000), α-tubulin (CST, #3873, 1:2,000), HIF-1α (CST, #3716, 1:1,000), β-actin (CST, #4970, 1:1,000), and GFP (CST, #2956, 1:1,000) were used. Horseradish peroxidase-conjugated anti-rabbit IgG (GE, NA9340, 1:2000) was used as a secondary antibody.

### Measurement of metabolites

The TA muscles or liver were immediately frozen in liquid nitrogen. The muscles were sectioned using a cryostat. Intramuscular lactate was analyzed using lactate-glo (Promega, J5021). Intramuscular pyruvate was analyzed using a pyruvate assay kit (BioVision, K609–100).

For LTT, 1 g/kg body weight (Figs. 4, C and D, 5B, 6B, and fig. S4B) or 1.5 g/kg body weight (figs. S5D and S6C) of lactate was intraperitoneally injected. Blood lactate or glucose levels at 5, 15, 30, 60, 120, and 180 min were measured using Lactate Pro2 (ARKRAY) or LAB Gluco (ForaCare Japan), respectively. For GTT, 3 g/kg body weight of glucose was intraperitoneally injected. Steady state levels of blood lactate were also measured using Lactate Pro2. To minimize the effect of prior physical activity, mice were transferred to individual cages and allowed to acclimate for a sufficient period to ensure a stable resting state before measurement.

For measurement of the NADH/NAD^+^ ratio, an NAD^+^/NADH Assay Kit (CELL BIOLABS, INC. MET-5014) was used.

### Stable isotope experiments

For the stable isotope experiments, 1 g/kg body weight ^13^C-lactate (M + 3) (Taiyo Nippon Sanso Corporation, 485926) was injected intraperitoneally into young or 18–20-month-old mice. Ten minutes after administration, the liver and TA muscles were immediately isolated and frozen in liquid nitrogen. ^13^C-lactate-derived metabolites were analyzed using Human Metabolome Technologies (HMT). Briefly, approximately 20–40 mg of frozen liver and TA muscle were placed in a homogenization tube along with zirconia beads. Then, 750 μL of 50% acetonitrile/Milli-Q water containing internal standards (H3304–1002, HMT) was added to the tube. The tissue was completely homogenized using a beads shaker. After centrifugation, the supernatant was centrifugally filtered through a 5-kDa cut-off filter (UltrafreeMC-PLHCC, HMT). The filtrate was evaporated to dryness under vacuum and reconstituted in 50 μL of Milli-Q water for metabolome analysis.

Metabolome analysis was conducted using HMT’s *F-SCOPE* package and capillary electrophoresis time-of-flight mass spectrometry (CE-TOFMS) as previously described (*59*, *60*). Peaks were extracted using MasterHands (Keio University, Tsuruoka, Yamagata, Japan) (*61*). Signal peaks corresponding to adduct ions and other product ions of known metabolites were excluded. The remaining peaks were annotated according to HMT’s metabolite database based on their *m/z* values and migration times (MTs). Isotopic ion annotation was performed using the calculated *m/z* values and MTs for each target compound. The areas of the annotated peaks were then normalized to internal standards to obtain relative levels of each metabolite, after which the metabolites were quantified based on the concentration of corresponding standard compounds. Finally, IsoCor (*62*), a Python package based on the method proposed by van Winden and coworkers (*63*), was used to correct isotopomer concentration altered by naturally abundant isotopes.

### NAD^+^ measurement by high-performance liquid chromatography (HPLC)

The amount of NAD^+^ was analyzed by HPLC as previously described with minor modifications (*64*). Briefly, the TA or GAS muscles, cells, or single muscle fibers were immediately frozen in liquid nitrogen. The frozen muscles, cells, or single muscle fibers were homogenized in 10% perchloric acid, and their lysate was neutralized with 3 M K_2_CO_3_, followed by NAD^+^ measurement using a Shimadzu HPLC system equipped with a SUPELCOSIL LC-18-T column (Merck). NAD^+^ levels per mg tissue weight or per well were calculated. For single muscle fibers, six single muscle fibers were analyzed for one measurement, and NAD^+^ levels per fiber were calculated.

### Single muscle fiber isolation and staining with fluorescence indicators

Isolation of single muscle fibers from the EDL muscle was performed as previously described (*14*, *65*). Brierly, EDL muscles were dissociated with 0.2% type 1 collagenase (Worthington, CLS-1)/Dulbecco’s Modified Eagle Medium (DMEM) at 37°C for 80–90 min. After gently pipetting the dissociated muscles, single muscle fibers were isolated using a glass pipette. For primary culture of muscle progenitor cells, isolated single muscle fibers were plated on Matrigel (CORNING, 356230)-coated dishes, and cultured in DMEM (high glucose, GlutaMAX, sodium pyruvate with phenol red, GIBCO, 10569010) supplemented with 20% fetal bovine serum (FBS), 2.5 ng/mL basic fibroblast growth factor and 1% penicillin-streptomycin at 37°C with 5% CO_2_.

Single muscle fibers were stained with fluorescence indicators immediately after isolation as previously described with minor modifications s*4*). Single muscle fibers were treated with 8 μM Fluo-4 a.m. (Dojindo, F311), 1 μM ER-tracker (ThermoFisher, E34250), 200 nM Mitotracker (ThermoFisher, M7510), 40 nM TMRM (Cayman, 21437), 50 nM Calcein-AM (Nacalai Tesque, 19177–14) or 5 μM pHrodo (ThermoFisher, P35372) in physiological salt solution (2.5 mM CaCl_2_, 140 mM NaCl, 5 mM KCl, 10 mM HEPES (pH 7.0), 1 mM MgCl_2_, and 10 mM glucose) for 30 min at room temperature to allow homogenous intracellular distribution of the dye. The stained fibers were analyzed using KEYENCE or Leica fluorescent microscope. The Fluo-4 normal and abnormal fibers from 26–28-month-old mice were separated using a Leica fluorescence stereomicroscope.

For absolute estimates of intracellular pH values in single muscle fibers, we used the Spexyte Intracellular pH Calibration Buffer Kit (Abcam, ab284662). In addition to 10 μM nigericin included in the kit, 10 μM valinomycin (Sigma-Aldrich, V0627) was added to equilibrate H^+^ and K^+^ ions. After pHrodo staining, the isolated single muscle fibers were incubated in the calibration buffer (pH 7.5, 7.0, 6.5 or 6.0), and pHrodo fluorescence was measured using a KEYENCE microscope.

Caffeine-induced increases in intracellular Ca^2+^ levels were measured as previously described, with modifications (*14*). Following staining with Fluo-4 a.m., Fluo-4 normal and abnormal fibers were isolated using a fluorescence stereomicroscope. These fibers were then treated with 10 mM caffeine, and the Fluo-4 fluorescence intensity was measured before and after treatment.

### Immunofluorescent analysis of single muscle fibers

Separated Fluo-4 normal and abnormal fibers were fixed with 4% paraformaldehyde/PBS solution. Fixed single fibers were treated with 0.1% Triton X/PBS for 15 min at room temperature, followed by blocking with 5% goat serum in 1% BSA/PBS for 15 min at room temperature. Single fibers were then stained with the first antibodies. Anti-RyR (DSHB, 34C, 1:4,000), anti-DHPR (NOVUS, NB300–542, 1:4000), or anti-SERCA (GENETEX, GTX22819, 1:4,000) in 1% BSA/PBS were used. After washing with PBS, single fibers were stained overnight with Alexa Fluor-conjugated secondary antibody in 1% BSA/PBS at 4°C. After washing, the stained single fibers were analyzed using KEYENCE.

### Electron microscopy of single muscle fibers

For transmission electron microscopy, isolated single muscle fibers were fixed using 2.5% glutaraldehyde (Fujifilm WAKO, 077–06271)/0.1 M phosphate buffer (pH 7.4). Single fibers were then fixed with 1% OsO_4_ in the same buffer. Fixed single muscle fibers were dehydrated using a graded series of ethanol and embedded in Epok812 (Oken Shoji). Ultrathin sections were prepared and stained with uranyl acetate and lead citrate. The specimens were examined using a HT7700 transmission electron microscope (Hitachi).

### AAV production

For the production of AAV-DJ, AAVpro 293 T cells (Takara Bio) were co-transfected with pAAV-DJ and pAdDeltaF6 in addition to pAAV-mCherry-sh-*firefly luciferase* (*fLuc*), −sh-*Ckmt2*, or -sh-*Perm1*. Three days after transfection, the transfected cells and their culture medium were collected and frozen and thawed three times. Cell debris was removed by centrifugation, and the supernatant was used as an AAV solution for in vitro analysis. For the production of AAV9, AAVpro 293 T cells were co-transfected with pAAV2/9n and pAdDeltaF6 in addition to pAAV-mCherry-sh-*fLuc*, −sh-*Ckmt2*, or -sh-*Perm1*. Three days after transfection, AAV was extracted using the AAVpro Cell & Sup. Purification Kit Maxi (Takara Bio). The AAV titers were determined using the AAV pro Titration Kit (for Real Time PCR) Ver. 2 (Takara Bio). AAV9-TBG-*c.a.HIF1*α and AAV9-TBG-*EGFP* were generated using Vector Builder.

### In vivo administration of AAV

For AAV9 administration, young mice were anesthetized, and 5 × 10^9^ vg or 7.5 × 10^9^ vg of AAV9-sh-*fLuc*, −sh-*Ckmt2*, or -sh-*Perm1* were injected into the TA or GAS muscles, respectively. A total of 1.5 × 10^11^ vg of AAV9-TBG-*c.a.HIF1a* or AAV9-TBG-*EGFP* were intravenously injected in 3–4-month-old and 19-month-old mice by a single dose via the tail vein.

### Cell culture and AAV infection in vitro

AAVpro 293 T and HepG2 cells were purchased from Takara Bio and JCRB, respectively. The cells were maintained with DMEM (Fujifilm Wako) supplemented with 10% FBS and 1% penicillin-streptomycin (Fujifilm Wako) at 37°C with 5% CO_2_. Isolated muscle fibers with primary muscle progenitor cells were cultured in DMEM (high glucose, sodium pyruvate, and GlutaMAX supplement; Thermo Fisher Scientific) supplemented with 20% FBS, 2.5 ng/mL of basic fibroblast growth factor (peprotech, 450–33), and 1% penicillin-streptomycin at 37°C with 5% CO_2_. Differentiation was induced by replacing the medium with DMEM supplemented with 10% FBS and 1% penicillin-streptomycin. For AAV infection, AAV was added at the induction of differentiation.

### Statistical analysis

All values are expressed as mean ± standard error of the mean (s.e.m.). The statistical significance of differences was assessed using Student’s t-test for comparison of two groups and one-way analyses of variance (ANOVA) with Tukey’s or Dunnett’s test for comparison of multiple groups. Two-way repeated-measures ANOVA with or without Sidak’s or Dunnett’s test was used for repeated measurement of two groups using Prism 8. Probabilities less than 5% (*, *P* < 0.05), 1% (**, *P* < 0.01), or 0.1% (***, *P* < 0.001) were considered statistically significant.
