# Supplementary material for: Impaired liver-muscle lactate metabolism causes sarcopenia via lactic acidosis in skeletal muscle in mice
Source: Sci Adv. 2026 Sep 4;12(36):eaeb4011. doi: 10.1126/sciadv.aeb4011 (PMC13544239; doi:10.1126/sciadv.aeb4011)
Supplement: Supplementary file 1 [file sciadv.aeb4011_sm.pdf]

Science Advances
AAAS

Supplementary Materials for

Impaired liver-muscle lactate metabolism causes sarcopenia via lactic acidosis in skeletal muscle in mice

Takahiro Eguchi *et al.*

Corresponding author: Naoki Ito, naoki.ito@ncgg.go.jp

Sci. Adv. **12**, eaeb4011 (2026)
DOI: 10.1126/sciadv.aeb4011

This PDF file includes:

Figs. S1 to S7
Table S1

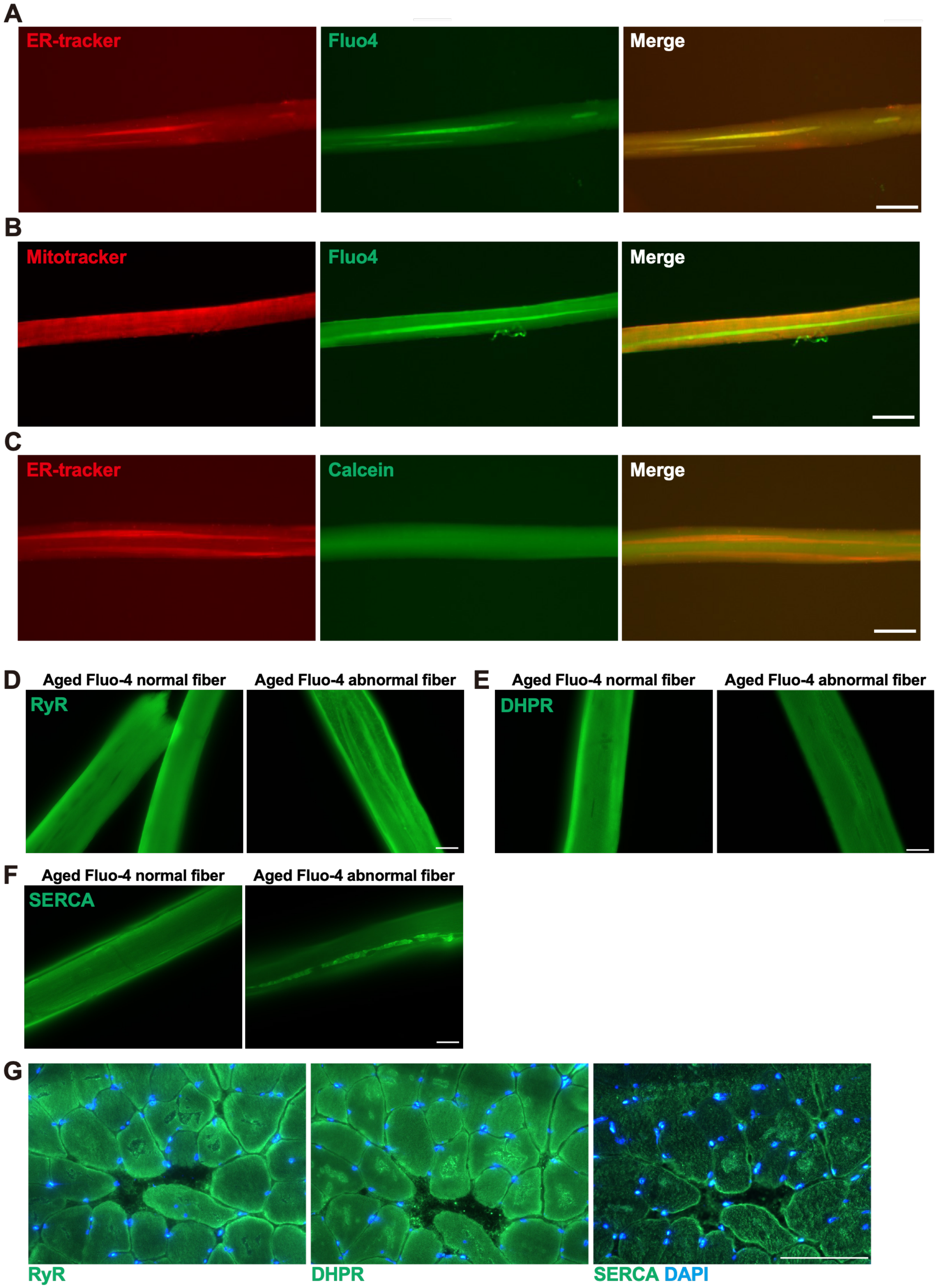

A
ER-tracker
Fluo4
Merge
B
Mitotracker
Fluo4
Merge
C
ER-tracker
Calcein
Merge
D
Aged Fluo-4 normal fiber
Aged Fluo-4 abnormal fiber
RyR
E
Aged Fluo-4 normal fiber
Aged Fluo-4 abnormal fiber
DHPR
F
Aged Fluo-4 normal fiber
Aged Fluo-4 abnormal fiber
SERCA
G
RyR
DHPR
SERCA DAPI

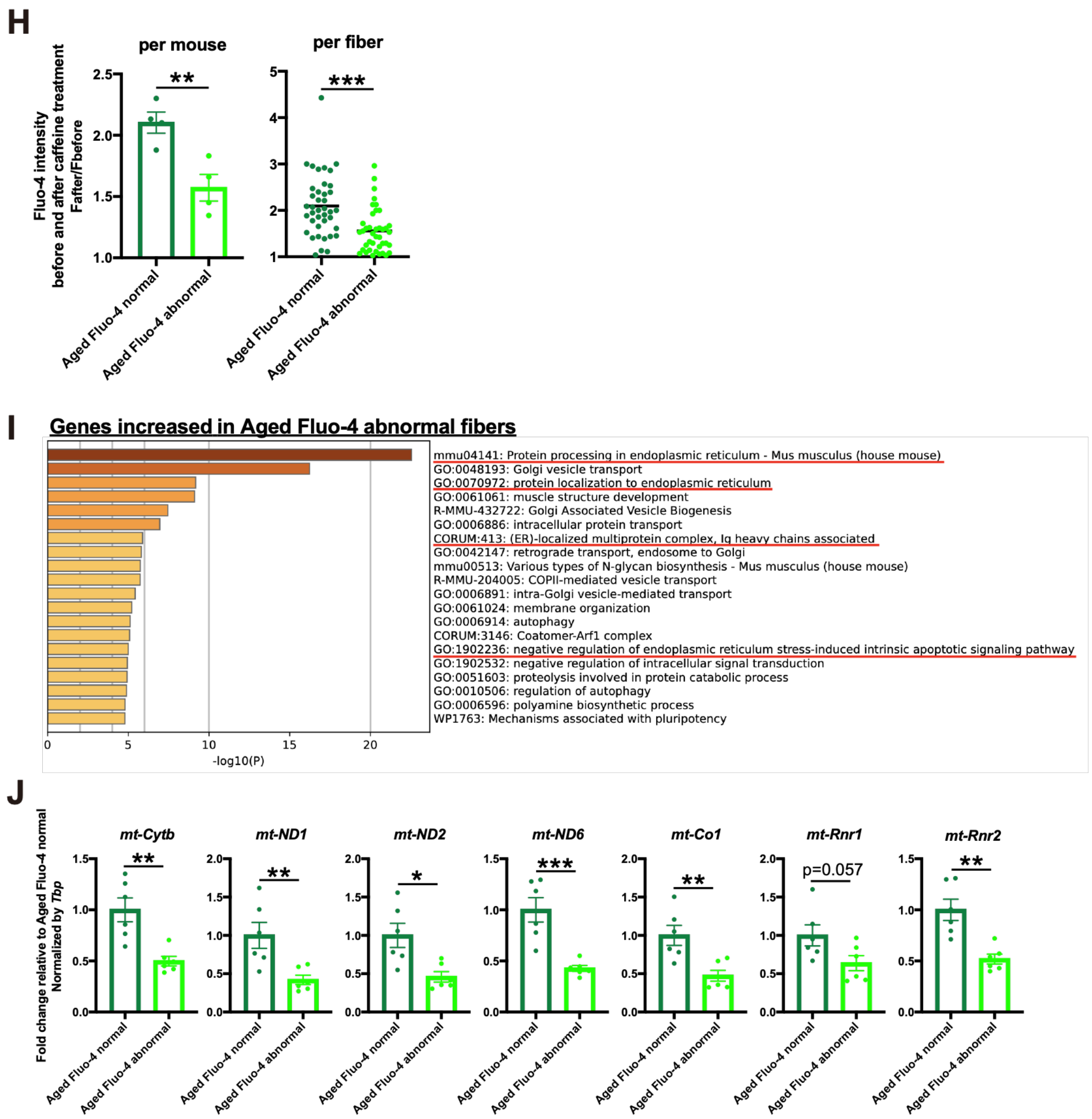
H
per mouse
per fiber
Fluo-4 intensity before and after caffeine treatment Fafter/Fbefore
**
***
Aged Fluo-4 normal
Aged Fluo-4 abnormal
I
Genes increased in Aged Fluo-4 abnormal fibers
mmu04141: Protein processing in endoplasmic reticulum - Mus musculus (house mouse)
GO:0048193: Golgi vesicle transport
GO:0070972: protein localization to endoplasmic reticulum
GO:0061061: muscle structure development
R-MMU-432722: Golgi Associated Vesicle Biogenesis
GO:0006886: intracellular protein transport
CORUM:413: (ER)-localized multiprotein complex, Ig heavy chains associated
GO:0042147: retrograde transport, endosome to Golgi
mmu00513: Various types of N-glycan biosynthesis - Mus musculus (house mouse)
R-MMU-204005: COPII-mediated vesicle transport
GO:0006891: intra-Golgi vesicle-mediated transport
GO:0061024: membrane organization
GO:0006914: autophagy
CORUM:3146: Coatomer-Arf1 complex
GO:1902236: negative regulation of endoplasmic reticulum stress-induced intrinsic apoptotic signaling pathway
GO:1902532: negative regulation of intracellular signal transduction
GO:0051603: proteolysis involved in protein catabolic process
GO:0010506: regulation of autophagy
GO:0006596: polyamine biosynthetic process
WP1763: Mechanisms associated with pluripotency
-log10(P)
J
Fold change relative to Aged Fluo-4 normal Normalized by Tbp
mt-Cytb
mt-ND1
mt-ND2
mt-ND6
mt-Co1
mt-Rnr1
mt-Rnr2
p=0.057
Aged Fluo-4 normal
Aged Fluo-4 abnormal

Fig. S1. Related to Figure 1, Identification and characterization of the Fluo-4 abnormal muscle fibers in aged mice.

(**A**-**C**) Representative fluorescence images of Fluo-4 and ER tracker (**A**), Fluo-4 and Mitotracker (**B**) and Calcein and ER-tracker (**C**) in single muscle fibers from 26-28-month-old aged mice. Bar: 100 μm. n=4. (**D**-**F**) Representative immunofluorescence images of RyR (**D**), DHPR (**E**), and SERCA (**F**) in the Fluo-4 normal and abnormal fibers. Bar: 10 μm. n=4. (**G**) Representative immunofluorescence images of RyR (left), DHPR (middle), and SERCA (right) in a transverse section of 18-20-month-old middle-aged mice. Bar: 100 μm. n=4. (**H**) Quantitative analysis of Fluo-4 intensity in the Fluo-4 normal and abnormal fibers before and after treatment with caffeine per mouse (left) and per fiber (right). n=4. (**I**) Gene ontology analysis of genes increased in the Fluo-4 abnormal fibers compared with the Fluo-4 normal fibers. (**J**) mRNA expression levels of mitochondrially-encoded genes in the Fluo-4 normal and abnormal fibers. n=6. *$P < 0.05$, **$P < 0.01$ and ***$P < 0.001$ by Student's t-test. Error bars indicate s.e.m.

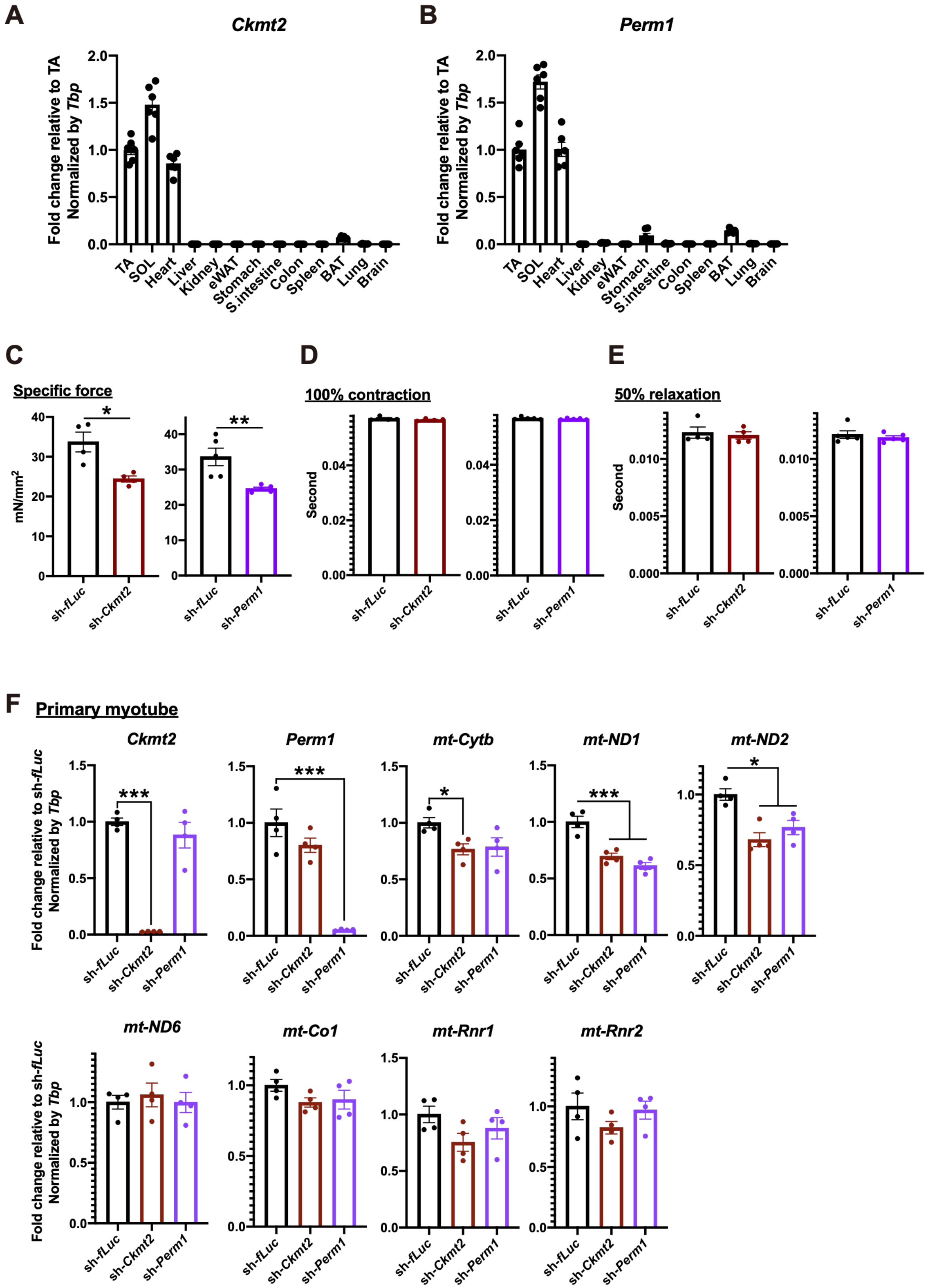
A
Ckmt2
Fold change relative to TA Normalized by Tbp
TA SOL Heart Liver Kidney eWAT Stomach S.intestine Colon Spleen BAT Lung Brain
B
Perm1
C
Specific force
mN/mm2
sh-fLuc
sh-Ckmt2
sh-Perm1
D
100% contraction
Second
E
50% relaxation
F
Primary myotube
Fold change relative to sh-fLuc Normalized by Tbp
Ckmt2
Perm1
mt-Cytb
mt-ND1
mt-ND2
mt-ND6
mt-Co1
mt-Rnr1
mt-Rnr2

Fig. S2. Related to Figure 2, *Ckmt2* and *Perm1* as the responsible genes for mitochondrially-encoded genes and age-associated declines in muscle force.
(**A**-**B**) mRNA expression levels of *Ckmt2* (**A**) and *Perm1* (**B**) in brain and peripheral tissues. n=5-6. (**C-E**) Specific force (**C**), 100% contraction time (**D**) and 50% relaxation time (**E**) measurements of *Ckmt2*- or *Perm1*-knockdown mice. n=4-5. (**F**) mRNA expression levels of *Ckmt2*, *Perm1* and mitochondrially-encoded genes in *Ckmt2*- or *Perm1*-knockdown primary myotubes. n=4. *$P < 0.05$, **$P < 0.01$ and ***$P < 0.001$ by Student's t-test for (**C**), or by one-way ANOVA with Dunnett's test for (**F**). Error bars indicate s.e.m.

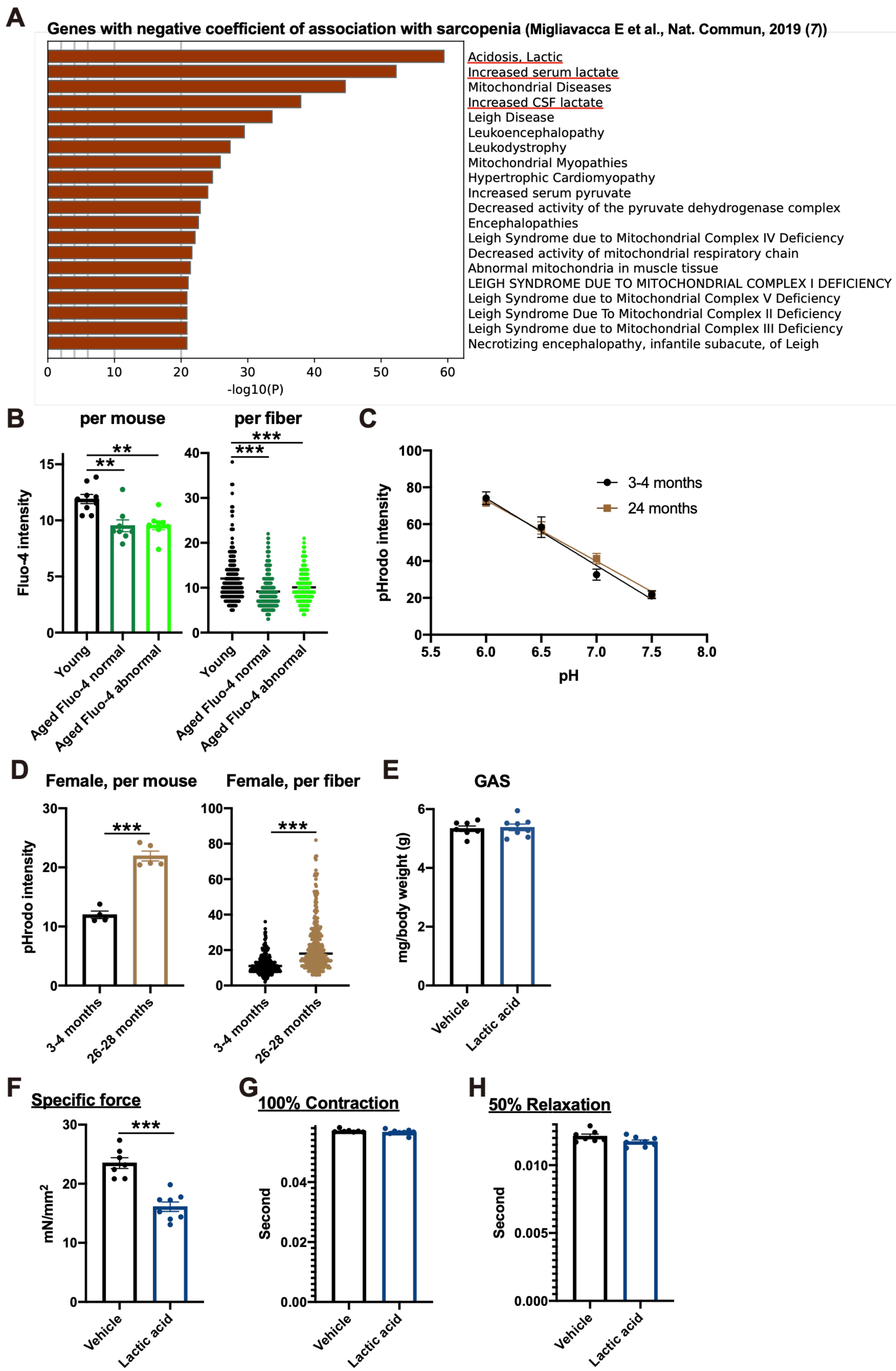

A
Genes with negative coefficient of association with sarcopenia (Migliavacca E et al., Nat. Commun, 2019 (7))
Acidosis, Lactic
Increased serum lactate
Mitochondrial Diseases
Increased CSF lactate
Leigh Disease
Leukoencephalopathy
Leukodystrophy
Mitochondrial Myopathies
Hypertrophic Cardiomyopathy
Increased serum pyruvate
Decreased activity of the pyruvate dehydrogenase complex
Encephalopathies
Leigh Syndrome due to Mitochondrial Complex IV Deficiency
Decreased activity of mitochondrial respiratory chain
Abnormal mitochondria in muscle tissue
LEIGH SYNDROME DUE TO MITOCHONDRIAL COMPLEX I DEFICIENCY
Leigh Syndrome due to Mitochondrial Complex V Deficiency
Leigh Syndrome Due To Mitochondrial Complex II Deficiency
Leigh Syndrome due to Mitochondrial Complex III Deficiency
Necrotizing encephalopathy, infantile subacute, of Leigh
-log10(P)
B
per mouse
per fiber
Fluo-4 intensity
Young
Aged Fluo-4 normal
Aged Fluo-4 abnormal
C
pHrodo intensity
3-4 months
24 months
pH
D
Female, per mouse
Female, per fiber
pHrodo intensity
3-4 months
26-28 months
E
GAS
mg/body weight (g)
Vehicle
Lactic acid
F
Specific force
mN/mm²
G
100% Contraction
Second
H
50% Relaxation
Second
Vehicle
Lactic acid

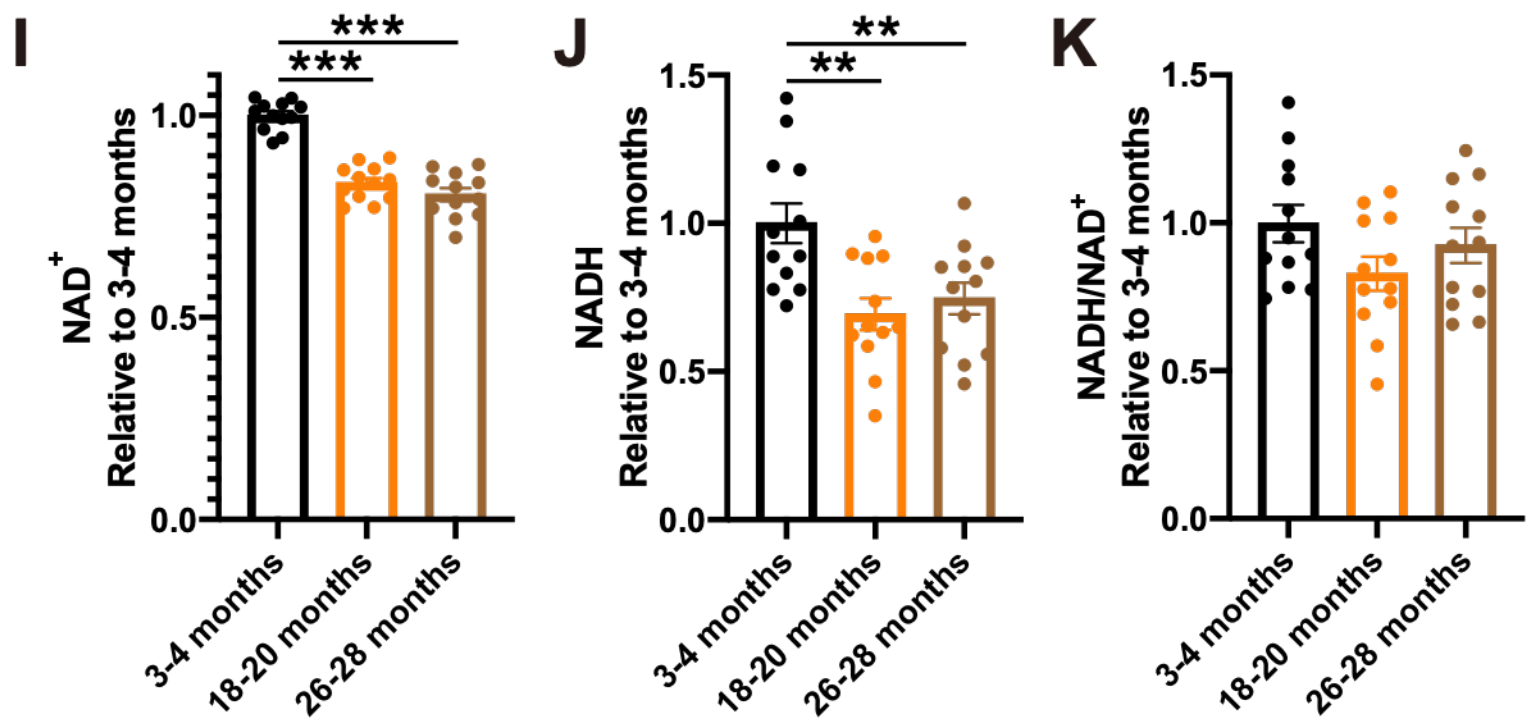

I
***
***
1.0
0.5
0.0
NAD+
Relative to 3-4 months
3-4 months
18-20 months
26-28 months
J
**
**
1.5
1.0
0.5
0.0
NADH
Relative to 3-4 months
3-4 months
18-20 months
26-28 months
K
1.5
1.0
0.5
0.0
NADH/NAD+
Relative to 3-4 months
3-4 months
18-20 months
26-28 months

Fig. S3. Related to Figure 3, Lactic acidosis in skeletal muscle as a cause of age-related decline in muscle force.

(**A**) Gene-disease network analysis of genes decreased in older adults with sarcopenia. (**B**) Quantitative analysis of Fluo-4 intensity in single muscle fibers from young, Fluo-4 normal, or Fluo-4 abnormal fibers from 26-28-month-old mice per mouse (left) and per fiber (right). n=8-9. (**C**) Absolute intracellular pH estimates in single muscle fibers from 3-4-month-old mice or 24-month-old mice. n=4. (**D**) Quantitative analysis of pHrodo intensity in single muscle fibers from young and 26-28-month-old aged female mice per mouse (left) and per fiber (right). n=4-5. (**E**) The GAS muscle weights of lactic acid-administered mice. n=7-8. (**F-H**) Specific force (**F**), 100% contraction time (**G**) and 50% relaxation time (**H**) measurements of lactic acid-administered mice. n=7-8. (**I-K**) Amounts of NAD^+ (**I**), NADH (**J**), or $NADH/NAD^+$ ratio (**K**) in the GAS muscle of 3-4-month-old young, 18-20-month-old middle-aged, and 26-28-month-old aged mice. n=12. $^{**}P < 0.01$ and $^{***}P < 0.001$ by Student's t-test for (**D**) and (**F**), or by one-way ANOVA with Dunnett's test for (**B**), (**I**), and (**J**). Error bars indicate s.e.m.

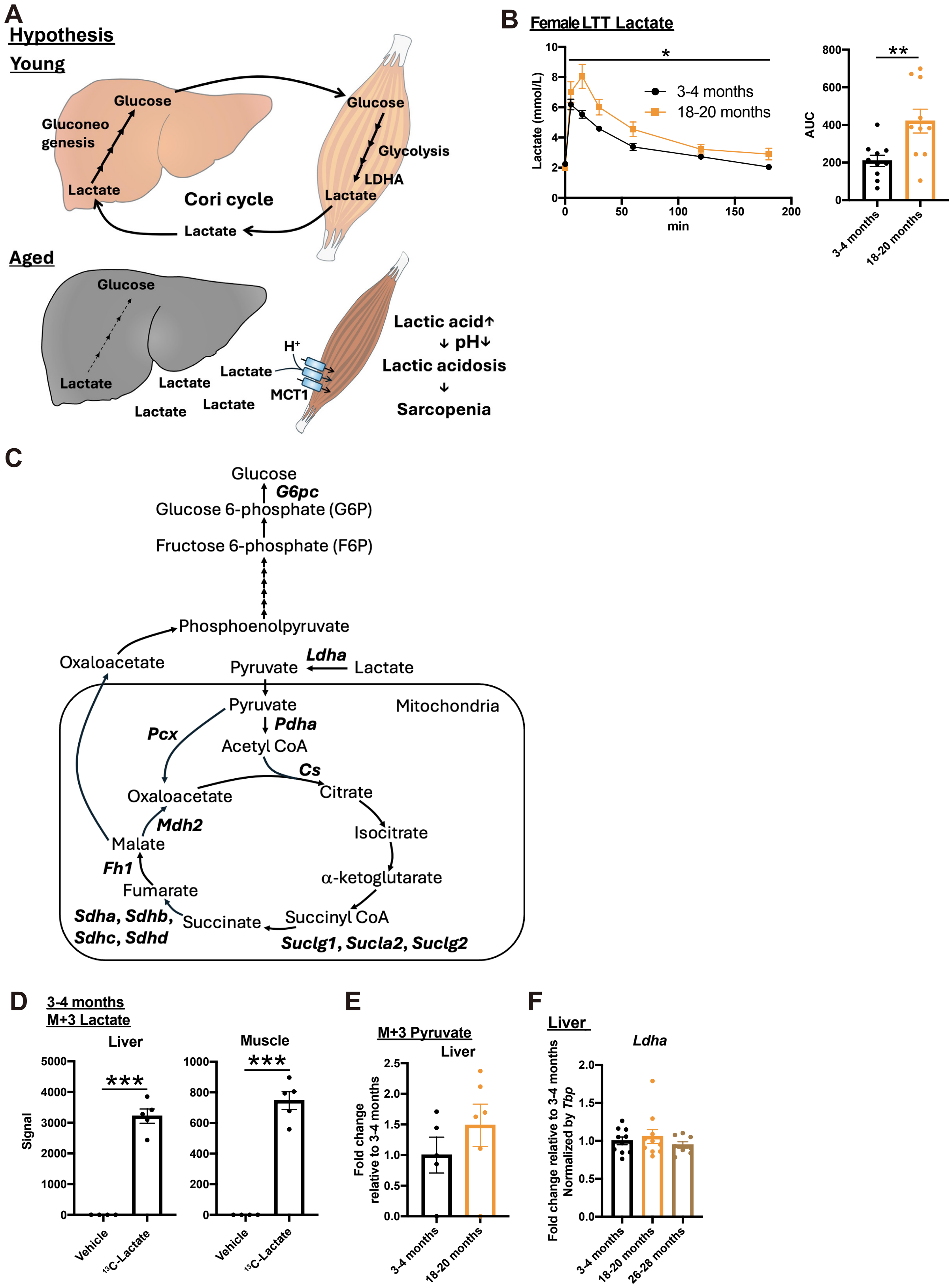
A
Hypothesis
Young
Glucose
Gluconeo genesis
Lactate
Glucose
Glycolysis
LDHA
Lactate
Cori cycle
Lactate
Aged
Glucose
Lactate
Lactate
Lactate
Lactate
Lactate
H+
MCT1
Lactic acid↑
↓ pH↓
Lactic acidosis
↓
Sarcopenia
B
Female LTT Lactate
Lactate (mmol/L)
min
3-4 months
18-20 months
AUC
3-4 months
18-20 months
C
Glucose
G6pc
Glucose 6-phosphate (G6P)
Fructose 6-phosphate (F6P)
Phosphoenolpyruvate
Oxaloacetate
Pyruvate
Ldha
Lactate
Pyruvate
Mitochondria
Pdha
Pcx
Acetyl CoA
Cs
Oxaloacetate
Citrate
Isocitrate
Mdh2
Malate
Fh1
α-ketoglutarate
Fumarate
Sdha, Sdhb, Sdhc, Sdhd
Succinate
Succinyl CoA
Suclg1, Sucla2, Suclg2
D
3-4 months
M+3 Lactate
Liver
Muscle
Signal
Vehicle
13C-Lactate
E
M+3 Pyruvate
Liver
Fold change relative to 3-4 months
3-4 months
18-20 months
F
Liver
Ldha
Fold change relative to 3-4 months Normalized by Tbp
3-4 months
18-20 months
26-28 months

G

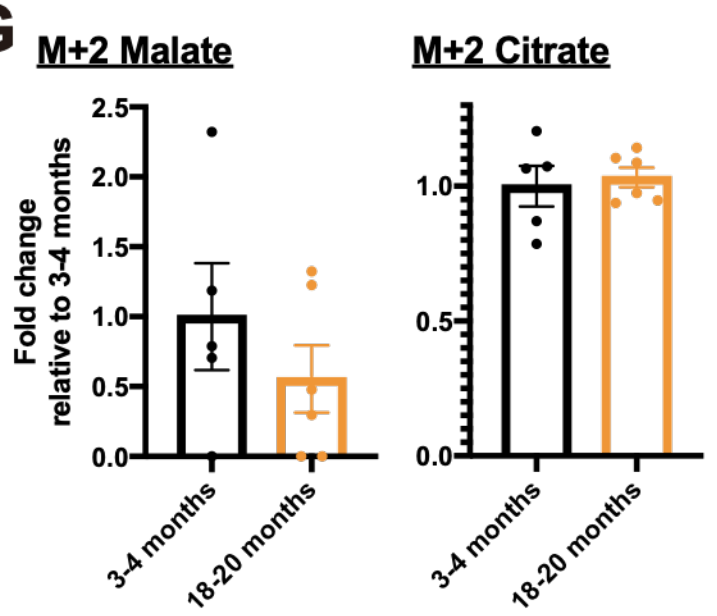
M+2 Malate
M+2 Citrate
Fold change relative to 3-4 months
3-4 months
18-20 months

H

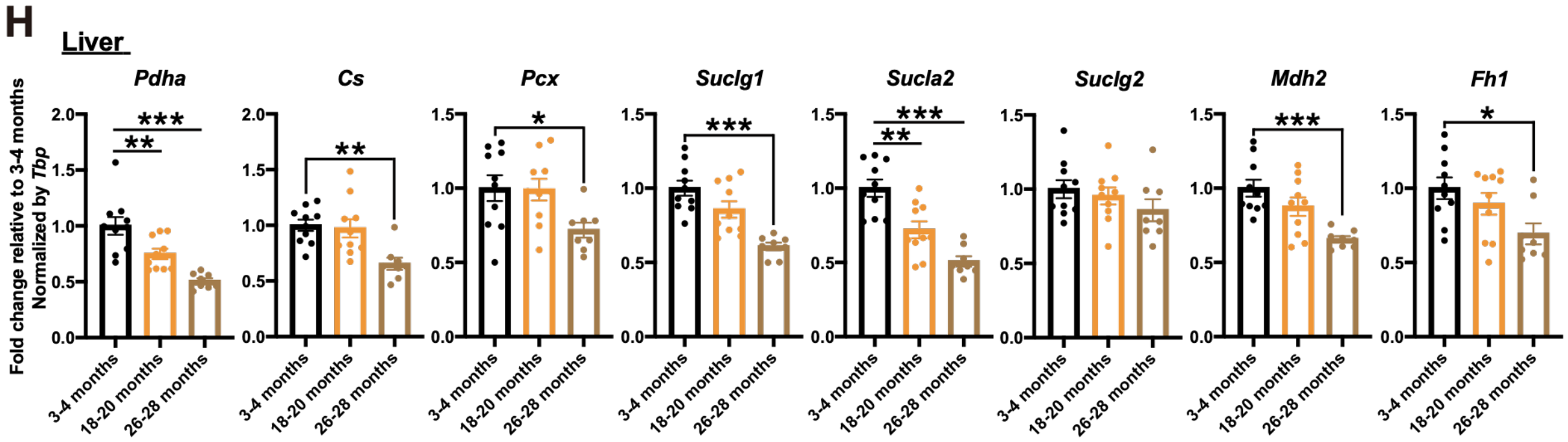
Liver
Pdha
Cs
Pcx
Suclg1
Sucla2
Suclg2
Mdh2
Fh1
Fold change relative to 3-4 months Normalized by Tbp
3-4 months
18-20 months
26-28 months

I

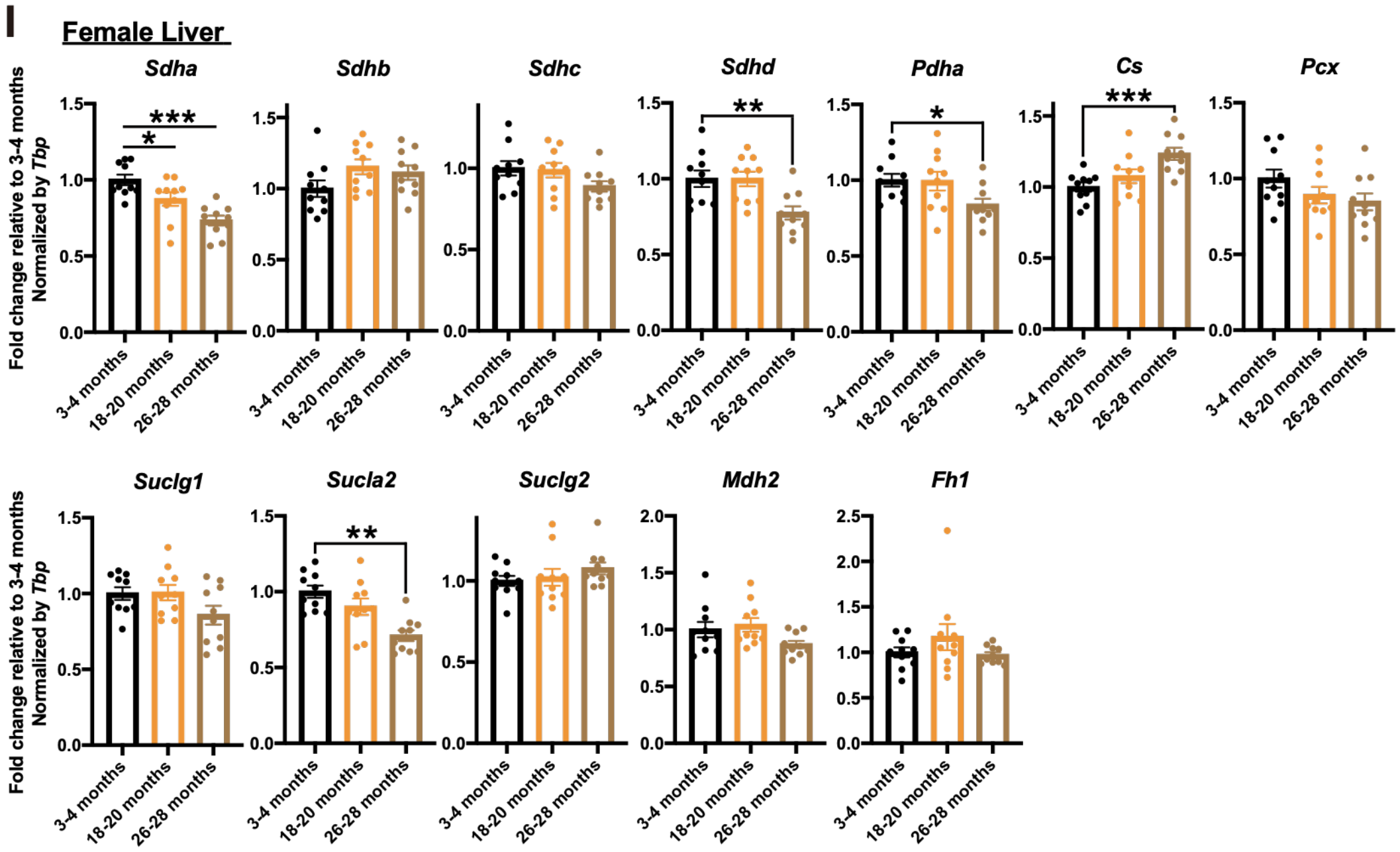
Female Liver
Sdha
Sdhb
Sdhc
Sdhd
Pdha
Cs
Pcx
Suclg1
Sucla2
Suclg2
Mdh2
Fh1
Fold change relative to 3-4 months Normalized by Tbp
3-4 months
18-20 months
26-28 months

J

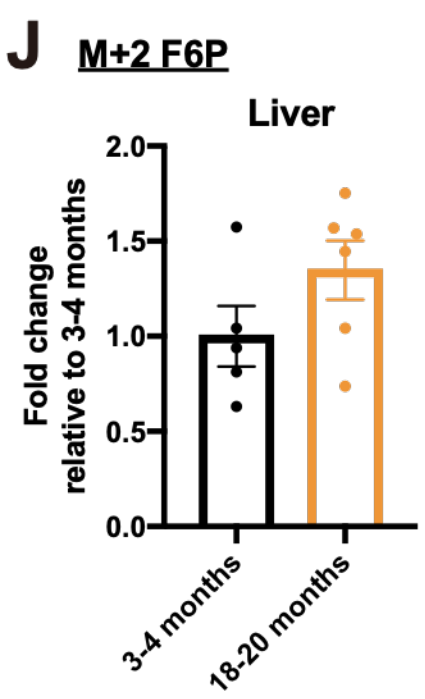
M+2 F6P
Liver
Fold change relative to 3-4 months
3-4 months
18-20 months

Fig. S4. Related to Figure 4, Decreased lactate processing capacity in the liver during aging. (**A**) Schematic hypothesis for the cause of lactic acidosis in skeletal muscle during aging. (**B**) Left: lactate tolerance test (LTT) in young or 18-20-month-old middle-aged female mice. Right: quantification of area under the curve (AUC) of LTT in young or 18-20-month-old female mice. n=10. (**C**) Graphical summary of the TCA cycle, gluconeogenesis, and their related genes. (**D**) Amount of ^{13}C-lactate (M+3) in the liver or muscle of young mice after vehicle or ^{13}C-lactate (M+3) administration. n=4-5. (**E**) Amount of ^{13}C-pyruvate (M+3) in the liver of young or 18-20-month-old mice after administration of ^{13}C-lactate. n=5-6. (**F**) mRNA expression levels of *Ldha* in the liver of young, 18-20-month-old, and 26-28-month-old aged mice. n=8-10. (**G**) Amount of ^{13}C-malate (M+2) or ^{13}C-citrate (M+2) in the liver of young or 18-20-month-old mice after administration of ^{13}C-lactate. n=5-6. (**H**) mRNA expression levels of *Pdha*, *Cs*, *Pcx*, *Suclg1*, *Sucla2*, *Suclg2*, *Mdh2*, and *Fh1* in the liver of young, 18-20-month-old, and 26-28-month-old mice. n=8-10. (**I**) mRNA expression levels of *Sdha*, *Sdhb*, *Sdhc*, *Sdhd*, *Pdha*, *Cs*, *Pcx*, *Suclg1*, *Sucla2*, *Suclg2*, *Mdh2*, and *Fh1* in the liver of young, 18-20-month-old, and 26-28-month-old female mice. n=10. (**J**) Amount of ^{13}C-F6P (M+2) in the liver of young or 18-20-month-old mice after administration of ^{13}C-lactate. n=5-6. $^{*}P < 0.05$, $^{**}P < 0.01$ and $^{***}P < 0.001$ by Student's t-test for (**B**, right) and (**D**), by one-way ANOVA with Dunnett's test for (**H**) and (**I**), or by two-way repeated-measures ANOVA for (**B**, left). Error bars indicate s.e.m.

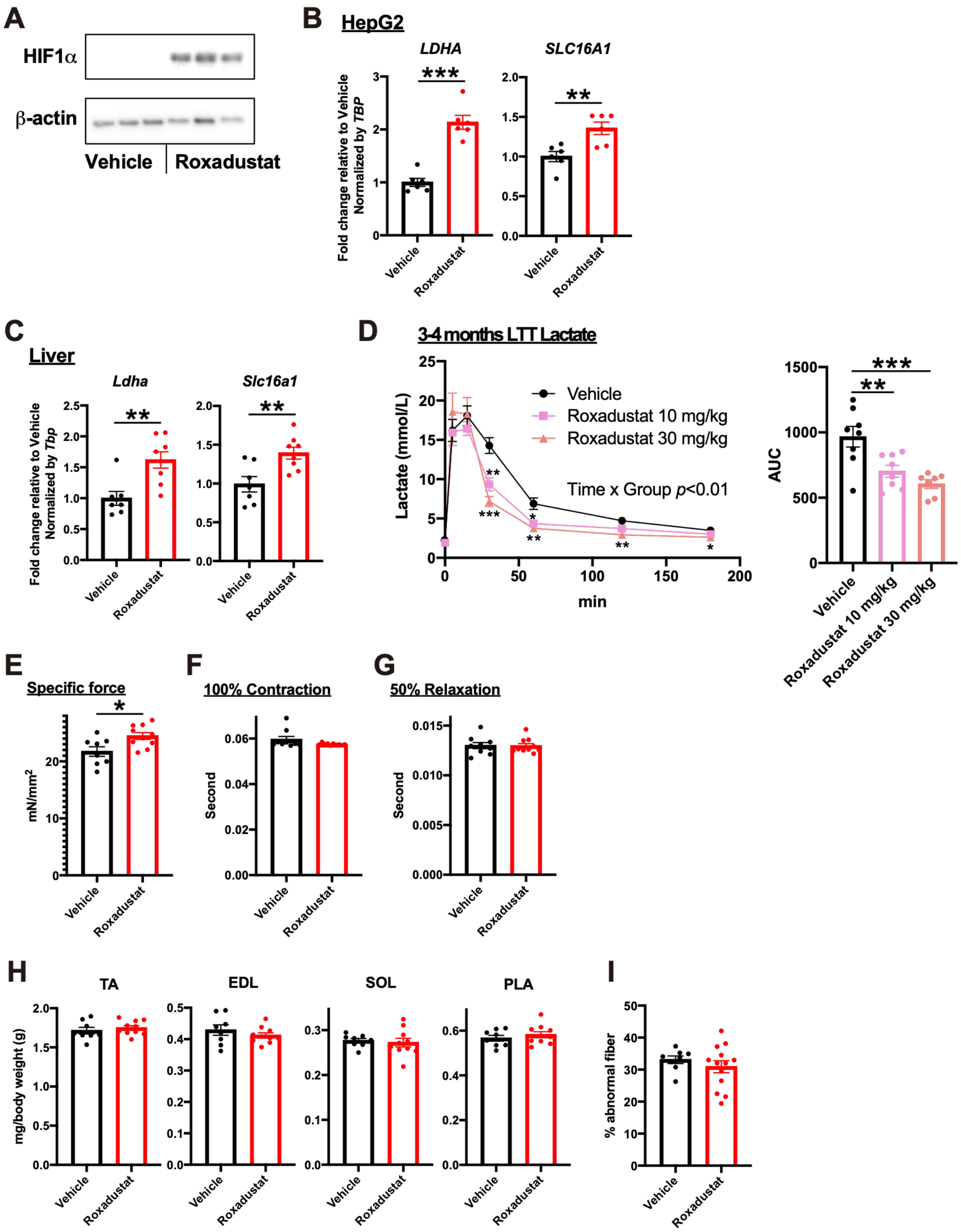
A
HIF1α
β-actin
Vehicle
Roxadustat
B
HepG2
LDHA
SLC16A1
Fold change relative to Vehicle Normalized by TBP
***
**
Vehicle
Roxadustat
C
Liver
Ldha
Slc16a1
Fold change relative to Vehicle Normalized by Tbp
**
**
Vehicle
Roxadustat
D
3-4 months LTT Lactate
Lactate (mmol/L)
Vehicle
Roxadustat 10 mg/kg
Roxadustat 30 mg/kg
Time x Group p<0.01
min
AUC
Vehicle
Roxadustat 10 mg/kg
Roxadustat 30 mg/kg
E
Specific force
mN/mm2
F
100% Contraction
Second
G
50% Relaxation
Second
H
TA
EDL
SOL
PLA
mg/body weight (g)
I
% abnormal fiber
Vehicle
Roxadustat

Fig. S5. Related to Figure 5, PHD inhibitor alleviates age-related decline in muscle force and lactic acidosis in skeletal muscle.

(**A**) Representative western blotting for HIF1α and β-actin in roxadustat-treated HepG2 cells. (**B**) mRNA expression levels of *LDHA* and *SLC16A1* in roxadustat-treated HepG2 cells. n=6. (**C**) mRNA expression levels of *Ldha* and *Slc16a1* in the liver of roxadustat-administered young mice. n=7-8. (**D**) Left: lactate tolerance test (LTT) in vehicle- or roxadustat (10 or 30 mg/kg)-administered young mice. Right: quantification of the area under the curve (AUC) of LTT in roxadustat-administered young mice. n=7-8. (**E-G**) Specific force (**E**), 100% contraction time (**F**) and 50% relaxation time (**G**) measurements of roxadustat-administered mice. n=9-10. (**H**) Hindlimb muscle weights of roxadustat-administered mice. n=8-10. (**I**) Quantitative analysis of the Fluo-4 abnormal fibers in roxadustat-administered mice. n=8-13. $^{*}P < 0.05$, $^{**}P < 0.01$, and $^{***}P < 0.001$ by Student's t-test for (**B**), (**C**), and (**E**), by one-way ANOVA with Dunnett's test for (**D**, right), or by two-way repeated-measures ANOVA with Dunnett's test for (**D**, left). Error bars indicate s.e.m.

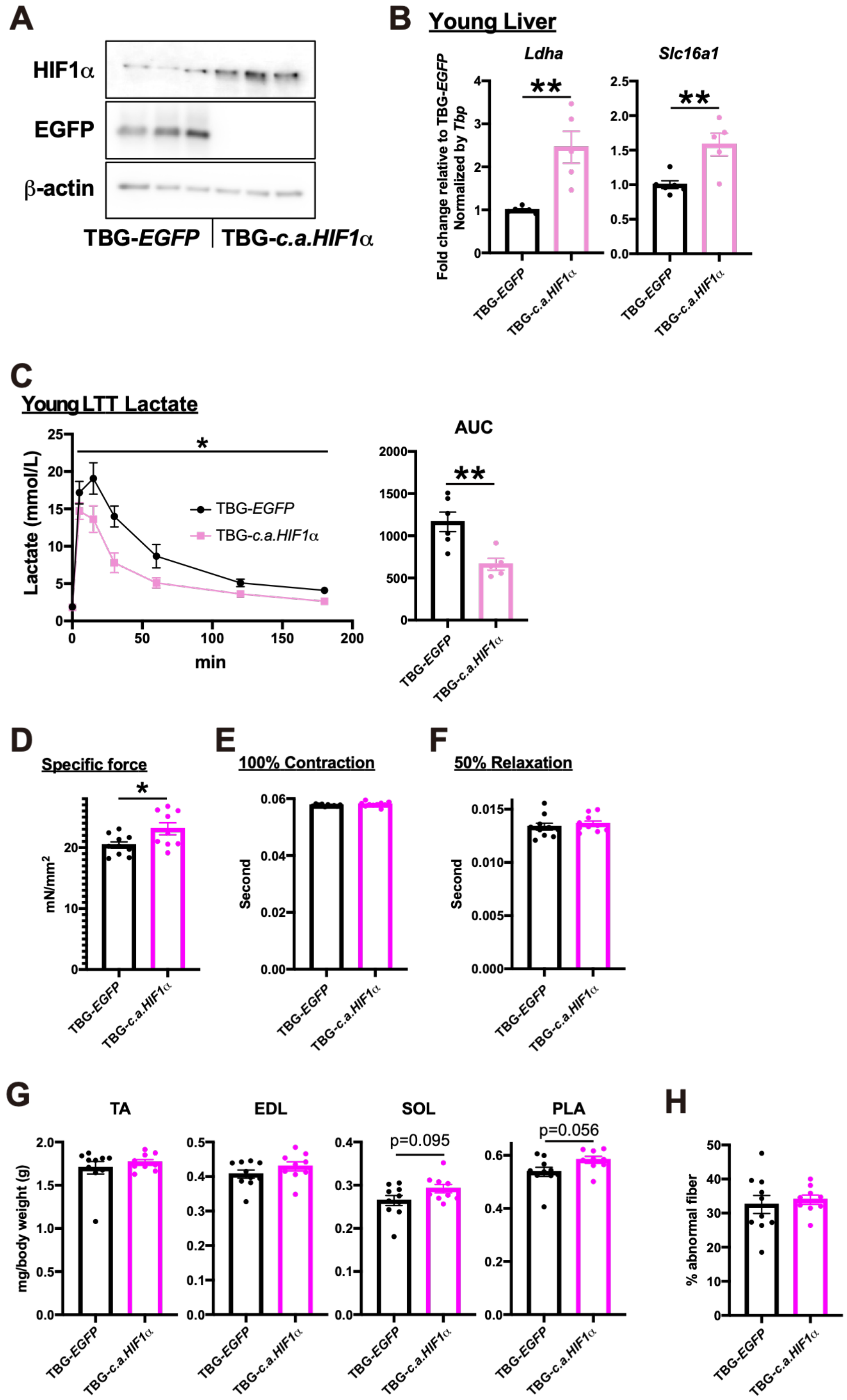

A
HIF1α
EGFP
β-actin
TBG-EGFP
TBG-c.a.HIF1α
B
Young Liver
Ldha
Slc16a1
Fold change relative to TBG-EGFP Normalized by Tbp
**
TBG-EGFP
TBG-c.a.HIF1α
C
Young LTT Lactate
Lactate (mmol/L)
min
*
AUC
**
D
Specific force
mN/mm²
*
E
100% Contraction
Second
F
50% Relaxation
Second
G
TA
EDL
SOL
PLA
p=0.095
p=0.056
mg/body weight (g)
H
% abnormal fiber

Fig. S6. Related to Figure 6, Liver-specific overexpression of constitutively active *HIF1α* alleviates age-related decline in muscle force and lactic acidosis in skeletal muscle.
(**A**) Representative western blotting for HIF1α and β-actin in liver-specific *EGFP*- or *c.a.HIF1α*-overexpressed mice. (**B**) mRNA expression levels of *Ldha* and *Slc16a1* in the liver of liver-specific *c.a.HIF1α*-overexpressed young mice. n=5-6. (**C**) Left: lactate tolerance test (LTT) in liver-specific *c.a.HIF1α*-overexpressed young mice. Right: quantification of area under the curve (AUC) of LTT in liver-specific *c.a.HIF1α*-overexpressed young mice. n=5-6. (**D-F**) Specific force (**D**), 100% contraction time (**E**) and 50% relaxation time (**F**) measurements of liver-specific *c.a.HIF1α*-overexpressed mice. n=10. (**G**) Hindlimb muscle weights of liver-specific *c.a.HIF1α*-overexpressed mice. n=9-10. (**H**) Quantitative analysis of the Fluo-4 abnormal fibers in liver-specific *c.a.HIF1α*-overexpressed mice. n=9-10. $^{*}P < 0.05$ and $^{**}P < 0.01$ by Student's t-test for (**B**), (**C**, right) and (**D**), or by two-way repeated-measures ANOVA for (**C**, left). Error bars indicate s.e.m.

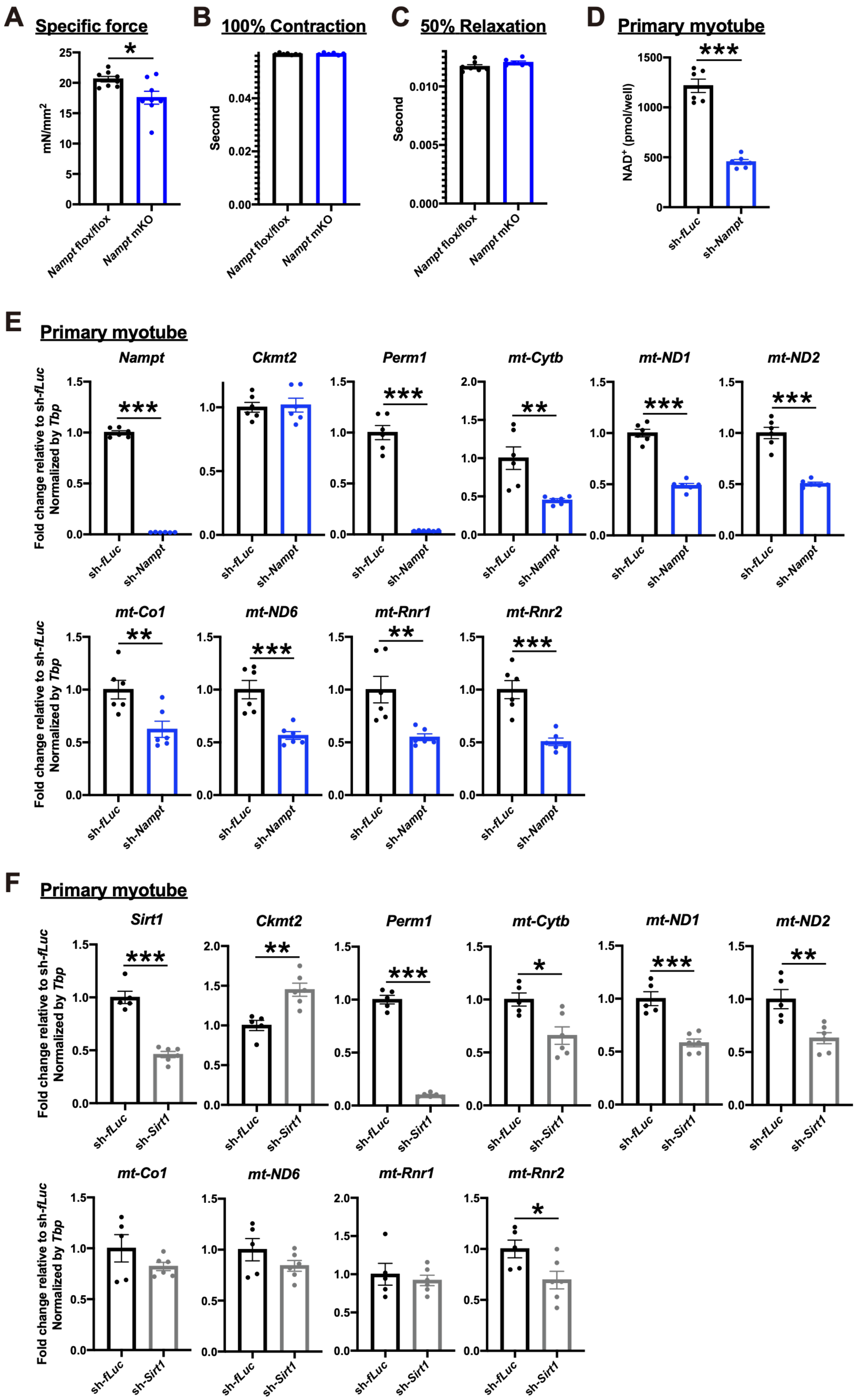
A
Specific force
mN/mm2
Nampt flox/flox
Nampt mKO
B
100% Contraction
Second
C
50% Relaxation
Second
D
Primary myotube
NAD+ (pmol/well)
sh-fLuc
sh-Nampt
E
Primary myotube
Nampt
Ckmt2
Perm1
mt-Cytb
mt-ND1
mt-ND2
mt-Co1
mt-ND6
mt-Rnr1
mt-Rnr2
Fold change relative to sh-fLuc
Normalized by Tbp
F
Primary myotube
Sirt1
sh-Sirt1

Fig. S7. Related to Figure 7, Skeletal muscle NAD$^+$ as an intermediary of lactic acidosis and age-related decline in muscle force.
(**A-C**) Specific force (**A**), 100% contraction time (**B**) and 50% relaxation time (**C**) measurements of *Nampt* mKO mice. n=6-7. (**D**) NAD$^+$ levels in *Nampt*-knockdown primary myotubes. n=6. (**E**) mRNA expression levels of *Nampt*, *Ckmt2*, *Perm1* and mitochondrially-encoded genes in *Nampt*-knockdown primary myotubes. n=6. (**F**) mRNA expression levels of *Sirt1*, *Ckmt2*, *Perm1*, and mitochondrially-encoded genes in *Sirt1*-knockdown primary myotubes. n=6. *$P < 0.05$, **$P < 0.01$ and ***$P < 0.001$ by Student's t-test. Error bars indicate s.e.m.

Ckmt2 forward 5'-acaagctctttcccccaagt-3'
Ckmt2 reverse 5'-cggagtttggcatagatggt-3'
Perm1 forward 5'-agatggccagagaggagtca-3'
Perm1 reverse 5'-acgcagctctggatgtcttt-3'
mt-Cytb forward 5'-tgagggggcttctcagtaga-3'
mt-Cytb reverse 5'-tagggccgcgataataaatg-3'
mt-ND1 forward 5'-acgcttccgttacgatcaac-3'
mt-ND1 reverse 5'-ggtggtactcccgctgtaaa-3'
mt-ND2 forward 5'-aacccacgatcaactgaagc-3'
mt-ND2 reverse 5'-gtacgatggccaggaggata-3'
mt-ND6 forward 5'-gcaaccctacacggaggtaa-3'
mt-ND6 reverse 5'-ccggttagaccaccaactgt-3'
mt-Co1 forward 5'-gcaaccctacacggaggtaa-3'
mt-Co1 reverse 5'-ccggttagaccaccaactgt-3'
mt-Rnr1 forward 5'-tcaaaggacttggcggtact-3'
mt-Rnr1 reverse 5'-tttgctgaagatggcggtat-3'
mt-Rnr2 forward 5'-gggataacagcgcaatccta-3'
mt-Rnr2 reverse 5'-attgggatgtcctgatccaa-3'
Ldha forward 5'-tggcagcctcttccttaaaa-3'
Ldha reverse 5'-gcggtgataatgaccagctt-3'
Slc16a1 forward 5'-tacacgtactgggcttgtgg-3'
Slc16a1 reverse 5'-ctccgctttctgttctttgg-3'
Slc16a3 forward 5'-acggctggtttcataacagg-3'
Slc16a3 reverse 5'-tcaggtcagtgaagccattg-3'
Sdha forward 5'-catggtcactagggctggtt-3'
Sdha reverse 5'-cacgacacccttctgtgatg-3'
Sdhb forward 5'-ctgccacaccatcatgaact-3'
Sdhb reverse 5'-ttaagccaatgctcgcttct-3'
Sdhc forward 5'-tgggaaccacagctaaggag-3'
Sdhc reverse 5'-acggacagtgccataggaag-3'
Sdhd forward 5'-ggtcagacccgcttatgtgt-3'
Sdhd reverse 5'-tggtgacaggtgaatgtgct-3'
G6pc forward 5'-gacctcctgtggactttgga-3'
G6pc reverse 5'-tggcaaagggtgtagtgtca-3'
Phda forward 5'-aagctaccgcactcgagaag-3'
Phda reverse 5'-ttgcaagattgctgttcacc-3'
Cs forward 5'-caacacctacggcttcccta-3'
Cs reverse 5'-ccgggtgtaattctcttcca-3'
Pcx forward 5'-agcaacggcttctgtcatct-3'
Pcx reverse 5'-gcacacaaccaagggaatct-3'
Suclg1 forward 5'-ttgtgcatggatgcaaagat-3'
Suclg1 reverse 5'-ctgcttctttgtccctttcg-3'

Sucla2 forward	5'-tggtgtaaaggaagcccaag-3'
Sucla2 reverse	5'-agttgacgatcccaccaaag-3'
Suclg2 forward	5'-ttgacatcgtcagagcgaac-3'
Suclg2 reverse	5'-ctgagagatcagggggatga-3'
Mdh2 forward	5'-aaaattgggcgaactcacac-3'
Mdh2 reverse	5'-cgcatactggacttgctgaa-3'
Fh1 forward	5'-aaaattgggcgaactcacac-3'
Fh1 reverse	5'-cgcatactggacttgctgaa-3'
Nampt forward	5'-agaagttgcaggaggggatt-3'
Nampt reverse	5'-gctgctggaacagaatagcc-3'
Nmnat1 forward	5'-tctacgagtccgatgtgctg-3'
Nmnat1 reverse	5'-tcttggtggacgagatgtca-3'
Nmnat3 forward	5'-tccagcagtttcagcacaac-3'
Nmnat3 forward	5'-ggaggtacttcacgctctgc-3'
Tbp forward	5'-cagcctcagtacagcaatcaac-3'
Tbp reverse	5'-taggggtcataggagtcattgg-3'
LDHA forward	5'-tgtgcctgtatggagtggaa-3'
LDHA reverse	5'-ctcaaccacctgcttgtgaa-3'
SLC16A1 forward	5'-gtggctcagctccgtattgt-3'
SLC16A1 reverse	5'-acagcattccacaatggtca-3'
TBP forward	5'-atgactcccggaatccctat-3'
TBP reverse	5'-gcctttgttgctcttccaaa-3'

Table S1. Oligonucleotides
